# Borg extrachromosomal elements of methane-oxidizing archaea have conserved and expressed genetic repertoires

**DOI:** 10.1038/s41467-024-49548-8

**Published:** 2024-06-26

**Authors:** Marie C. Schoelmerich, Lynn Ly, Jacob West-Roberts, Ling-Dong Shi, Cong Shen, Nikhil S. Malvankar, Najwa Taib, Simonetta Gribaldo, Ben J. Woodcroft, Christopher W. Schadt, Basem Al-Shayeb, Xiaoguang Dai, Christopher Mozsary, Scott Hickey, Christine He, John Beaulaurier, Sissel Juul, Rohan Sachdeva, Jillian F. Banfield

**Affiliations:** 1 Innovative Genomics Institute, University of California, Berkeley, CA USA; 2 Oxford Nanopore Technologies Inc, New York, NY USA; 3 Department of Environmental Science, Policy and Management, University of California, Berkeley, CA USA; 4 Microbial Sciences Institute, Yale University, New Haven, CT USA; 5 Deptartment of Molecular Biophysics and Biochemistry, Yale University, West Haven, CT USA; 6 Institut Pasteur, Université de Paris cité, Unit Evolutionary Biology of the Microbial Cell, Paris, France; 7 Centre for Microbiome Research, School of Biomedical Sciences, Queensland University of Technology (QUT), Translational Research Institute, Woolloongabba, QLD Australia; 8 Biosciences Division, Oak Ridge National Laboratory, Oak Ridge, TN USA; 9 Department of Microbiology, University of Tennessee, Knoxville, TN USA; 10 Biomedicine Discovery Institute, Monash University, Clayton, VIC Australia; 11 Department of Earth and Planetary Science, University of California, Berkeley, CA USA; 12 Present Address: Department of Environmental Systems Sciences, ETH Zurich, 8092 Zurich, Switzerland

**Keywords:** Environmental microbiology, Archaea

## Abstract

Borgs are huge extrachromosomal elements (ECE) of anaerobic methane-consuming “*Candidatus* Methanoperedens” archaea. Here, we used nanopore sequencing to validate published complete genomes curated from short reads and to reconstruct new genomes. 13 complete and four near-complete linear genomes share 40 genes that define a largely syntenous genome backbone. We use these conserved genes to identify new Borgs from peatland soil and to delineate Borg phylogeny, revealing two major clades. Remarkably, Borg genes encoding nanowire-like electron-transferring cytochromes and cell surface proteins are more highly expressed than those of host *Methanoperedens*, indicating that Borgs augment the *Methanoperedens* activity in situ. We reconstructed the first complete 4.00 Mbp genome for a *Methanoperedens* that is inferred to be a Borg host and predicted its methylation motifs, which differ from pervasive TC and CC methylation motifs of the Borgs. Thus, methylation may enable *Methanoperedens* to distinguish their genomes from those of Borgs. Very high Borg to *Methanoperedens* ratios and structural predictions suggest that Borgs may be capable of encapsulation. The findings clearly define Borgs as a distinct class of ECE with shared genomic signatures, establish their diversification from a common ancestor with genetic inheritance, and raise the possibility of periodic existence outside of host cells.

## Abstract (web summary)

Borgs are huge extrachromosomal elements associated with anaerobic methane-consuming archaea. Here, the authors use nanopore sequencing to identify new Borgs from peatland soil and their hosts, uncover their DNA modifications and activities, and elucidate the evolutionary relationships of Borgs.

## Introduction

Of all genetic entities in the biosphere, extrachromosomal elements of archaea may be the least well understood. Existing databases of archaeal viruses, plasmids and mini-chromosomes are limited (NCBI Virus, 556 viruses, Jun-02-2023 and NCBI Plasmids, 481 plasmids; www.ncbi.nlm.nih.gov/genome/browse#!/overview/) and generally taxonomically restricted to better known archaeal groups, such as the orders Sulfolobales and Halobacteriales. For many major groups of archaea, not even a single ECE has been described, and those that affiliate with uncultivated archaea are only inferred from metagenomic sequence information.

We recently described a seemingly new type of archaeal ECE that is not classifiable as virus or plasmid^1^. They are unusually large, up to 1.1 Mbp in length, and have linear genomes terminated by kilobase-scale long inverted terminal repeats (ITR). The genes are encoded on two replichores of very uneven length, with essentially all genes on each replichore carried on a single strand. These ECEs are predicted to replicate in *Methanoperedens* archaea based on sequence similarity, phylogeny, shared abundance-based co-occurrence patterns, and CRISPR targeting^1^. Because of their propensity to assimilate large numbers of genes from organisms, especially but not limited to their host *Methanoperedens*, they were named “Borgs”^1^. To date, their sequences have only been recovered from environmental metagenomic datasets from terrestrial subsurface ecosystems and, interestingly, often encode components of key metabolic processes such as anaerobic methane oxidation and nitrogen fixation^1^.

When new and unusual ECEs are recovered from metagenomic datasets, they may be questioned because methods for curation of short read assemblies into complete genomes are poorly understood and rarely undertaken^2^. Curation is important because short read assemblies are often fragmented and without a complete genome it is impossible to rule out the possibility that the sequence is, for example, a virus for which the structural proteins were not recovered, or an ECE that is integrated (e.g., pro-plasmid). Lack of complete or near-complete genomes also precludes the identification of genes that are universally present and the comparison of genome architectures. Here, we used long-read sequences from Oxford Nanopore Technologies that can span repeated regions to validate the overall topologies of some of our short-read-derived complete Borg genomes. Nanopore-derived sequence information was combined with information from more accurate and deeply sequenced short Illumina reads and contigs to reconstruct additional complete and near-complete genomes.

Anaerobic methane-oxidizing archaea of the genus *Methanoperedens* are known primarily from bioreactor co-cultures^3,4^. To date, only one complete *Methanoperedens* genome has been reported^5^ and it does not host Borgs. We obtained one complete and one near-complete genome for two *Methanoperedens* which are both predicted to host Borgs. These genomes enabled the comparison of Borg and host genetic repertoires as well as gene expression patterns. Overall, our results greatly increase sampling of the genomes and genetic repertoires of Borgs, establish that previously reported Borg characteristics are conserved, and reveal a mostly syntenous set of conserved marker genes that could be used to identify new Borgs and define their phylogeny.

## Results

### Evaluation of existing complete Borg genome sequences

**Fig. 1:**
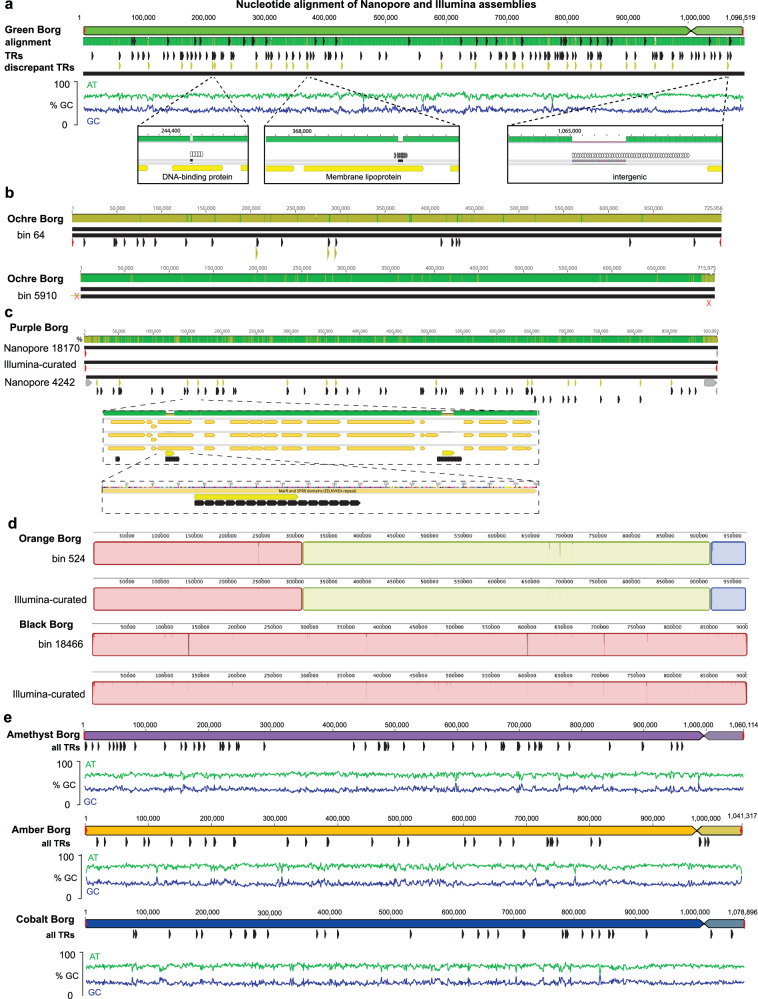
Confirmation of Borg genome architecture, including characteristic features reported based on Illumina assemblies, and overviews of new Borg genomes. **a** Alignment of nanopore and Illumina assemblies for Green Borg. The nanopore Green Borg genome differs from the manually curated Illumina genome almost exclusively in the number of units repeats in the TR regions (discrepant TRs shown as yellow marks). All TR regions are shown in black, expanded TR regions come to light in the nanopore genome and are shown as discrepant TRs in yellow. **b** Comparison of the published Ochre genome manually curated from Illumina reads to two versions based on the assembly of nanopore reads. The first relatively low-accuracy nanopore sequence (bin 64) confirms the overall topology of the Illumina-based genome, including the presence of inverted terminal repeats. The second nanopore sequence has high base accuracy but contained a ~10 kbp chimeric start (much higher GC content, trimmed from the 5910 sequences used in the alignment) and had a low accuracy terminal region (marked X). **c** The Illumina-based Purple Borg genome aligned to two nanopore-derived sequences providing overall validation. Also shown are examples of genic and intergenic regions where nanopore unit repeat count differs. **d** The overall topology of genomes for Orange and Black Borgs, including terminal inverted repeats, were confirmed using nanopore-assembled sequences; differences were again localized in TR regions. **e** Overview of three new curated complete genomes recovered from nanopore assemblies. The linear genomes are composed of two replichores and terminated by inverted terminal repeats shown in red.

The majority of previously reported Borg genomes were derived from soil sampled from a seasonal wetland in Lake County, northern California, and all were reconstructed using Illumina sequences^1,6^. We performed long-read nanopore sequencing on a subset of the same samples to evaluate the accuracy of the manually curated Illumina-based complete genomes reconstructed from these samples and to recover new Borg genomes. We sequenced both native DNA and DNA amplified with multiple displacement amplification (MDA), which served as a negative control for methylation calling and had more accurate basecalls, due to fewer signal current disruptions caused by modified bases. Obviously, erroneously assembled regions, such as chimeric joins that were often accompanied by a substantial jump in GC content and mirror image artifacts that resulted from the multiple displacement amplification process, were removed. The corrected nanopore-assembled sequences validate the genomes of Green, Ochre, Purple, Orange, and Black Borgs (Fig. 1a–d, Table 1). In most cases, the near-complete curated nanopore-derived sequences extend into, thus confirming, the ITR.

**Table 1:** Detailed statistics on available Borg genomes

Borg	bp	Status	GC (%)	TR regions	TR regions/100 kbp	TR region length	TR region (%)	TR unit count	TR intergenic	TR in ORF
Green	1094519	complete	33.6	76	6.94	10223	0.93	379	46	30
**Cobalt**	1078846	curated	31.8	41	3.80	4552	0.42	221	26	15
**Amethyst**	1060114	complete	34.1	65	6.13	10377	0.98	429	32	33
**Amber**	1041317	complete	32.7	45	4.32	6045	0.58	259	11	34
Orange	974068	complete	32.4	53	5.44	5675	0.58	246	33	20
Brown	937932	complete	32.2	51	5.44	5913	0.63	259	28	23
**Ruby**	922385	curated	33.5	62	6.72	6967	0.76	351	35	27
Purple	918293	complete	32.0	66	7.19	6701	0.73	326	42	24
Black	901883	complete	32.2	57	6.32	6128	0.68	289	33	24
**Iris**	845347	curated	31.9	35	4.14	4878	0.58	215	27	8
Sky	763094	complete	33.0	32	4.19	3889	0.51	206	21	11
Ochre	725447	complete	33.0	23	3.17	2108	0.29	92	12	11
Red	685823	complete	32.2	23	3.35	3181	0.46	173	11	12
**Viridian**	673463	curated	34.3	28	4.16	3110	0.46	152	15	13
Lilac	661708	complete	32.0	61	9.22	6733	1.02	299	33	28
Rose	623782	complete	32.8	20	3.21	2132	0.34	98	8	12
**Emerald**	613519	curated	34.0	19	3.10	3955	0.64	88	1	18

Newly reported genomes from this study are highlighted in bold. The other 10 genomes were published previously^5^.

The ~1.1 Mbp Green Borg is the largest Borg genome recovered to date. After removal of an obviously chimeric start (not supported by mapped Illumina reads), the nanopore genome aligns completely with the published Illumina-based genome (Fig. 1a). The Green Borg genome was reported to have a slightly more complex genome architecture than all other Borgs due to a switch in coding strand over a small region of the large replichore (Supplementary Fig. 1). This is verified by the nanopore assembly (Fig. 1a). Essentially all disagreements with the published genome involved a larger than expected number of repeat units in the tandem repeat (TR) regions. This is unsurprising, as some TR regions are longer than the Illumina read lengths. However, the number of TR units in TR regions is often variable within populations^6^, so a combination of deep Illumina and some longer nanopore reads may be best used to define the TR regions.

### Improved and new Borg genomes reveal conserved features

We used nanopore read assemblies to improve the quality of known Borg genomes and to reconstruct new genomes. Nanopore reads were assembled, mirror image sequence blocks and chimeras were removed and then sequencing errors were corrected by automated short- and long-read polishing methods followed by careful manual curation using Illumina reads and assembled Illumina contigs. Removal of single base pair errors, such as in homopolymers, is important as these can interrupt gene predictions. Ultimately, we reconstructed two new complete Borg genomes (Amethyst and Amber) and one essentially complete genome (Cobalt Borg, curated to 1.08 Mbp and into the ITR) (Fig. 1e). In addition, four new near-complete genomes for Iris, Emerald, Ruby, and Viridian Borgs were curated into single long contigs. Emerald and Viridian genomes include both replichores (Table 1, Supplementary Fig. 2).

Amethyst Borg is related to Blue Borg, the genome of which is currently available only in draft form. Borg-like contigs with consistently high coverage and low GC content were joined and aligned to the new Amethyst genome to generate a concatenated ~876 kbp Blue Borg sequence comprising the expected single strand coding of two replichores and termination by ITR.

All Borg genomes are linear and have long ITR, with an overall low GC content (~32–35%). However, there are local regions with elevated GC content. Some of these have high sequence similarity and very similar GC content to genes of *Methanoperedens*. There is near perfect single strand coding on each of the replichores, and many of the encoded proteins are specific to Borgs. The majority of genes (63%) lack a taxonomic affiliation (12,875 of 20,469). On average, 19.6% were taxonomically classified as archaeal (Supplementary Data 1). All newly reported genomes have the expected GC skew, consistent with replication initiation from the termini (Supplementary Fig. 1).

All Borg genomes harbor pervasive nucleotide tandem repeats that are located in intergenic regions and within open reading frames (Table 1). Approximately half of these TRs occur in genes, where they essentially always introduce amino acid repeats that generally confer local intrinsic disorder, as reported by us previously^6^. Representative examples in Amber and Amethyst Borg are TRs located in multiheme cytochromes (MHC) (Supplementary Fig. 3), which often harbor amino acid tandem repeats^6^. These regions may provide sites for post-translational modifications, mediate complex formation, or bind small molecules^6,7^. Outstanding is Emerald Borg, because it only has one TR in an intergenic region, and eighteen within ORFs. Overall, the well-defined Borg genomes have consistent features, supporting their assignment to a specific class of extrachromosomal elements.

### Borgs have a core genome that suggests a shared evolutionary origin

We used the 17 complete and near-complete genomes (Table 1) to identify conserved genes. Protein family clustering of all 20,280 Borg proteins revealed that 88.0% of Borg proteins have at least one homolog in another Borg (Supplementary Data 2). A comparison of shared protein subfamilies revealed that Black and Brown-Borg share the highest number of protein subfamilies, and Emerald and Red Borgs are most distantly related (Supplementary Fig. 4a, b).

Of the 2598 Borg protein subfamilies, 107 are present in all Borgs and thus make up the core Borg proteome (Supplementary Data 2, Supplementary Data 3). This core encompasses 40 subfamilies representing marker proteins (*n* = 1 in each Borg), 22 subfamilies representing near marker proteins (*n* = 1 in 16/17 Borgs), and 45 subfamilies representing multicopy marker proteins (multiple homologs in each Borg). Only ~18% (6/40) of the Borg marker proteins have homologs in the complete *Methanoperedens* genome.

**Fig. 2:**
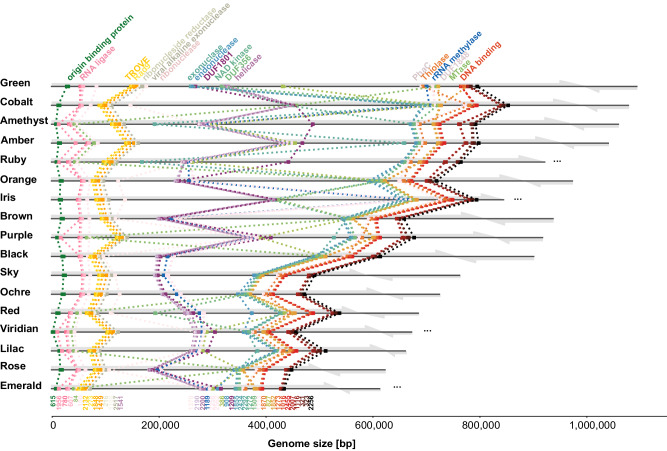
Borg protein family clustering defines relatedness and reveals syntenous localization of marker genes on Borg genomes. Complete Borg genomes are listed in order of decreasing length, with near-complete genomes included in order of the assembled lengths of the large replichore. Each replichore is represented by a gray arrow. Dots indicate the locations of the 40 marker genes (*n* = 1 in every Borg). Functional annotations are shown at the top, and subfamily numbers are at the bottom of the figure.

Mapping the 40 Borg marker genes onto each Borg genome revealed a fairly consistent localization of each subfamily gene across the entire large replichore, implying a shared genomic backbone (Fig. 2). This analysis also demonstrated that subsets of conserved genes are co-localized, suggesting a functional relationship and motivating a more general analysis of shared Borg proteins.

### Core Borg proteins for replication, nucleotide processing, cell decoration, signaling, carbon metabolism and redox

**Fig. 3:**
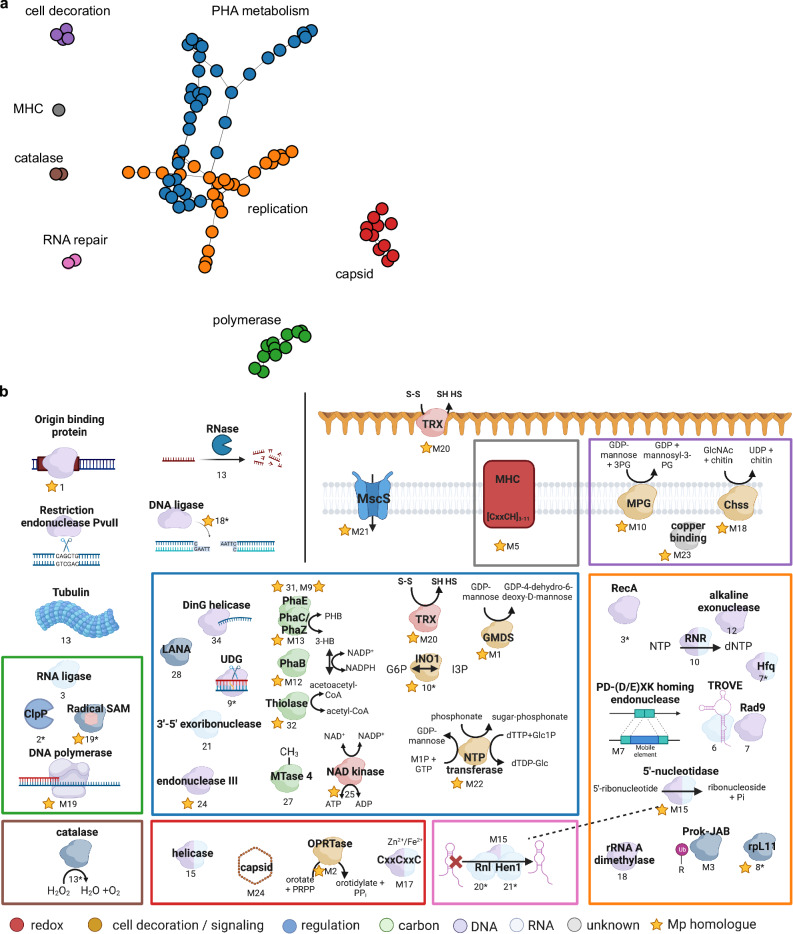
Protein network of core Borg genes and their putative functions. **a** Core Borg proteins were plotted in distinct clusters based on their consistent colocalization 10 genes up- or downstream of each other (*n* > 5). **b** Core Borg proteins with functional annotations are shown. Proteins of the same cluster are grouped together in colored boxes. Numbers below objects indicate that they are either marker proteins (numbers), near marker proteins (number and *), or multicopy proteins (number preceded by the letter M). More detailed descriptions of the objects are listed in Supplementary Data 3. **b** was created with BioRender.com.

To investigate the hypothesis that co-localized genes are functionally related we grouped together marker, near marker, and multicopy proteins based on colocalization, considering these core proteins to be co-localized if they occur within ten genes in a minimum of five Borg genomes. This generated eight core clusters comprising 84 of the 107 core subfamilies (Fig. 3a). We then examined the putative functions of the encoded core proteins, leveraging a combination of sequence-based functional annotations and structure-based (AlphaFold2 (AF2)^8^) homology predictions (Fig. 3b). We found structural matches for five core proteins that did not have any sequence-based annotations. One of these matches the DarG toxin (subfam2258, PDB match 5M3E) of the toxin-antitoxin system DarTG. DarG expression affects DNA replication by catalyzing the reversible ADP-ribosylation of thymidines in ssDNA^9^. We speculate that this protein could be involved in regulating Borg or host replication. The other four have credible matches to viral capsid proteins (see below).

Some core proteins are predicted to be involved in replicating Borg genomes. The 18 (including Blue) Borg genomes have a marker protein encoded relatively close to the start of the large replichore that has functional domains that match those of the Herpesvirus OBP (PF02399, Herpes_ori_bp). OBP binds to the origin of replication and initiates the formation of a pre-replication complex^10^. Emerald is the first reported Borg to encode a typical replication initiation protein, cdc6, situated just five genes downstream from the OBP. The second core gene cluster encoded on Borg genomes (green in Fig. 3a, b) includes a DNA polymerase B, accompanied by a YspA-related gene (subfam0180 and subfam1707) that could function as a sensor of nucleotide, nucleotide-derived ligands, or nucleic acids^11^. The DNA polymerase is highly conserved in Borgs and likely involved in replication.

The co-localized core proteome encodes many genes whose predicted functions imply roles in sensing and responding to DNA damage. For example, the second core gene cluster includes an RNA ligase capable of repairing breaks in nicked DNA:RNA and RNA:RNA. Interestingly, the predicted Borg protein structure is most akin to the RNA ligase from *Escherichia* virus T4 (PDB match 1s68, RMSD = 2.72), known for repairing tRNA breaks induced by a host protein following phage infection^12^. This may indicate the need for Borgs to protect their tRNA (Borgs encode 5-23 tRNAs per genome) and the host tRNA from degradation by the host archaeon. This genomic context also includes a radical SAM protein, a homing endonuclease, and a ClpP protease (PDB match 5vz2) which could be degrading defective and misfolded proteins; altered function of this protein has been shown to affect virulence and infectivity of pathogens^13^.

The third cluster (orange in Fig. 3a, b) features a TROVE domain protein, likely involved in RNA-binding and degradation^14^, and Rad9, which may monitor and respond to DNA damage. Following this is a cluster potentially involved in DNA processing/modification (Supplementary Fig. 5), including a DNA methylase (subfam0757 and subfam0999 where the DNA methylase is fused to an intein) that may introduce N^4^ cytosine-specific or N^6^ adenine-specific DNA methylations (4mC or 6mA). No close homologs of this methylase exist in any *Methanoperedens* (highest amino acid identity is 51%).

Further suggesting roles in sensing and responding to damage, the cluster represented by blue dots in Fig. 3 includes a protein (21) with remarkable structural homology to the 3’−5’ DNA exonuclease Cap18 from *Escherichia coli* (PDB match 7t2s, RMSD = 0.96)^15^. This is part of the bacterial immune cyclic oligonucleotide-based anti-phage signaling (CBASS) system, yet no other part of the system has been identified in Borgs and *Methanoperedens*. Also present is an endonuclease III (24, PDB match 1p59, RMSD = 1.66) that may function as a DNA glycosylase with repair activity specific for oxidized pyrimidine lesions in duplex DNA^16^. Other small clusters encode a putative Pnkp1–Rnl–Hen1 RNA repair complex (pink clusters), which is required to repair ribotoxin-cleaved RNA^17^, a manganese catalase potentially involved in hydrogen peroxide detoxification (brown clusters), or cell decoration machinery (purple cluster and members of the orange cluster). One cluster (red in Fig. 3a, b) comprises seven protein subfamilies, which encode a Rad3-related helicase (15), capable of unwinding dsDNA or DNA:RNA^18^, and possibly contributing to nucleotide excision repair, and an orotate phosphoribosyltransferase (OPRTase), putatively involved in pyrimidine synthesis.

A subset of the core families is predicted to have roles in metabolism. Strikingly, all Borgs encode the polyhydroxyalkanoate (PHA) pathway (blue in Fig. 3) that likely functions in PHA degradation, given the domain annotations and structural homology to a hydrolase (PhaC, PDB match 3om8). The PHA pathway also exists in many *Methanoperedens* genomes and has been implicated in PHA synthesis^5^.

**Fig. 4:**
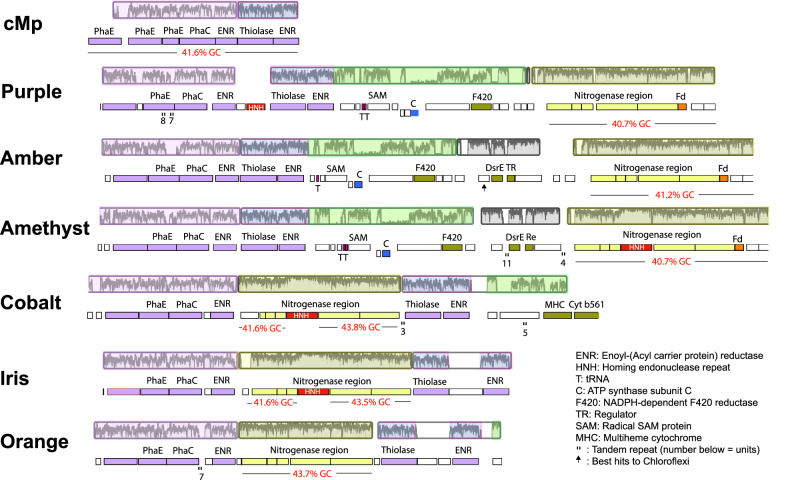
A region encoding genes involved in N_2_ fixation has been inserted within the region encoding for PHA metabolism in the Cobalt, Iris, and Orange Borg genomes. This nitrogenase-related region occurs downstream in three other Borg genomes. The Borg nitrogenase-related genes have elevated GC contents that match that of the complete *Methanoperedens* cMp genome. Graphs indicating nucleotide-level sequence identity across the Borg and *Methanoperedens* genomes are shown above the coding strand and gene annotation information; highlight colors indicate the largely syntenous blocks.

Nine Borgs encode a *nif* gene cluster with elevated GC content and sequence similarity to clusters in *Methanoperedens*. In Green, Ochre and Sky Borgs, and *Methanoperedens*, the *nif* cluster is located far distant from the PHA cluster. In Cobalt, Iris, and Orange Borgs it interrupts the PHA cluster, and in Purple, Amethyst, and Amber Borg genomes, the *nif* cluster is located just downstream of the PHA cluster (Fig. 4). The colocalization of nitrogenase and PHA clusters suggests that they could be functionally connected. We speculate that the reducing power (NAD(P)H) from PHA degradation could be harnessed by nitrogenase to fuel nitrogen fixation and potentially even concomitant H_2_ formation^19^. Also within this cluster is an NAD kinase, which regulates cellular NADP(H) levels, a crucial redox carrier in the PHA pathway. The NifH protein phylogeny groups the Amethyst sequence within one clade of *Methanoperedens* sequences, and the Ochre sequence within another clade of *Methanoperedens* sequences, whereas most other Borg NifH form a Borg-specific clade. Elsewhere, Green and Black Borgs encode an isolated NifH-like protein closely related to sequences found in *Methanoperedens*. These patterns point to repeated lateral transfer of *nif* genes into Borgs, and sequences grouping with those of *Methanoperedens* may suggest Borg-host associations.

We identified 207 Borg-encoded multiheme cytochromes (MHCs that have ≥3 CxxCH) with up to 33 classical heme-binding motifs (Supplementary Data 4). Interestingly, MHCs represent one of the largest multicopy subfamilies (subfam1158, subfam1369, subfam2060, subfam2491), but given their very divergent sequences, only one subfamily emerged in the protein network analysis (gray Fig. 3a, b). Cobalt Borg encodes a maximum of 19 different MHCs. The Borg-encoded MHCs likely augment the ability of *Methanoperedens* to transfer electrons that are liberated during methane oxidation onto extracellular electron acceptors.

### Soil distribution patterns as a clue to *Methanoperedens* - Borg linkages

The pairing of Borgs and hosts is a topic we attempted to address experimentally without success (see Methods). However, Borg and *Methanoperedens* relative abundances increase with soil depth, and Borg and *Methanoperedens* types vary with soil depth (e.g., Rose in shallower soils; Black and Brown in medium-depth soils, Viridian in the deeper soils; Supplementary Fig. 6), providing a clue to Borg-host associations. For example, when organized by presence but without strong reliance on abundance data, the patterns support the previous prediction that Black Borg replicates in *Methanperedens* “bMp”^1^, and hints that Orange and Ochre Borgs replicate in *Methanoperedens* “cMp”. Supporting the Orange host prediction, the Orange Borg exhibits more homologs (21%, 258/1,243) with cMp than other Borgs (Supplementary Data 5).

We used the abundance data to seek statistically significant correlations and found support for the replication of Black Borg in bMp. Interestingly, Brown Borg abundances are correlated with those of Black Borg, suggesting that it may also replicate in bMp. These Borgs also show significant correlations (>0.995) with five additional *Methanoperedens* species. A weaker correlation (0.961) was identified for Sky Borg and *Methanoperedens*_80cm_43_82 (Supplementary Data 6). The lack of strong correlations for the majority of Borgs from the wetland may be due to (a) variable Borg to *Methanoperedens* genome copy number (as suggested previously;^1^), (b) the same Borg resident in multiple different hosts, or (c) Borg DNA derived from outside of *Methanoperedens* cells.

Clarifying these possibilities, we note that Orange Borg is 72 times more abundant than the most abundant co-occurring *Methanoperedens* in 1.05 m deep soil (Supplementary Fig. 6, Supplementary Data 7). Such a high copy number seems very unlikely, especially if there is a stable association, for example if this Borg existed as a plasmid-like element in a single host. Even if Orange Borg was present in every single type of coexisting *Methanoperedens* cell, the abundance ratio is still 7.5 to 1. This also seems unlikely, especially given two other abundant Borgs (Ochre and Cobalt) co-occur in this sample. Simply comparing the totals for all Borgs and all *Methanoperedens* in the 1.05 m sample yields a predicted average genome copy number ratio of 8.8 to 1. These observations raise the possibility that some Borg DNA derives from cell-free genomes. Given no strong indication of DNA degradation from the ends of their linear genomes (based on read mapping), we consider the possibility that the Borg DNA can exist external to the cell, and that it is protected, possibly via encapsulation.

### Borgs encode a putative capsid and replication machinery akin to eukaryotic viruses

**Fig. 5:**
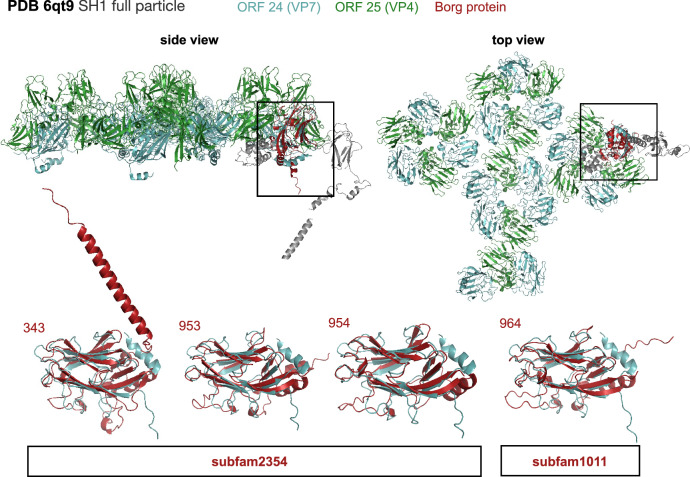
Structural model of putative capsid proteins from Orange Borg and superimposed on the structural matches from an archaeal virus. Structures were predicted with AF2, best structural matches were identified in the PDB using foldseek and visualized and superimposed in PyMOL. Orange Borg proteins and their corresponding locus tags/gene numbers are shown in red. The major capsid proteins of the *Haloacrcula hispanica* virus SH1 are ORF 24 (cyan) and ORF 25 (green), minor capsid proteins are shown in gray. The SH1 full particle structure was downloaded from PDB (6qt9).

The AF2-generated protein structure prediction analysis suggested that four core proteins without any sequence-inferred annotations had best structural matches with capsid proteins from *Haloarcula hispanica* virus SH1 (PDB 6qt9), and from the archaeal extremophilic internal membrane-containing *Haloarcula californiae* icosahedral virus 1 (HCIV-1, PDB 6h9c) (Fig. 5, Supplementary Data 8)^20,21^. Like Borgs, these viruses have linear dsDNA genomes terminated by inverted repeats, yet they are only ~30 kbp. The Borg-encoded putative capsid proteins open the possibility of Borg encapsulation at a point in their existence.

CheckV^22^ (v1.0.1) detected between 11 to 21 viral-like genes per Borg genome (Supplementary Data 9), whereas geNomad^23^ (v1.5.1) detected 39 possible provirus regions and no plasmid genes (Supplementary Data 10). One credible match found in all Borgs and many linear viruses is the Borg marker subfam2517. This protein functionally and structurally matches a viral recombinase/alkaline exonuclease (Supplementary Data 11, Supplementary Fig. 5), which is crucial in recombination and ultimately replication of viruses with linear dsDNA^24^. Manual inspection of the annotated Borg proteins revealed that 48 individual Borg proteins, including a marker protein (subfam0386), are annotated as related to KSHV latency-associated nuclear antigen, which promotes Herpesvirus persistence^25^. Interestingly, a protein with similar domain annotations (Herpes_alk_exo) is involved in the replication of eukaryotic Herpesviruses (HSV) and is required for the production of viral DNA^26^. HSV encodes seven essential replication proteins, three of which may correspond to the Borg marker proteins DNA PolB and the PCNA-like Rad9, as well as a marker helicase C (Supplementary Data 11). All Borgs encode the OBP replication initiation protein similar to that from HSV. Moreover, all Borgs encode one copy of a tubulin-like protein (subfam2487). Similar cytoskeletal proteins in HSV form nanotubes that facilitate cell-to-cell contacts, and are essential for efficient viral spreading and replication^27^. Notably, HSV has a double-stranded DNA genome, some of which are linear, and many genomes (≥92) feature numerous tandem repeats. Together, these observations indicate shared features with this (much smaller) eukaryotic virus type and bring to light indications that Borgs share substantial replication-related machinery with diverse viruses.

### Borgs and hosts have distinct DNA modifications

We used Illumina reads to correct a small number of base errors in a nanopore-assembled sequence and generated a circular, complete 4,003,944 Mbp *Methanoperedens* genome (cMp). This genome is substantially larger than the only other complete ~3.3 Mbp genome of *M. nitroreducens*^5^, which does not host Borgs^28^. The cMp genome is predicted to host Orange and Ochre Borgs (Supplementary Fig. 6), and we also detected Black, Purple, Cobalt, and Ruby Borgs in this sample (SR-VP_9_9_2021_72_4B_1.05 m).

From the same soil sample we also reconstructed a near-complete, 2.43 Mbp *Methanoperedens* genome (bMp) that is on four contigs. This genome was previously suggested to represent the host for Black Borg based on statistically significant abundance-based co-occurrence patterns (with Borg: host abundance ratios ranging from 0.5 to 8×;^1^).

**Fig. 6:**
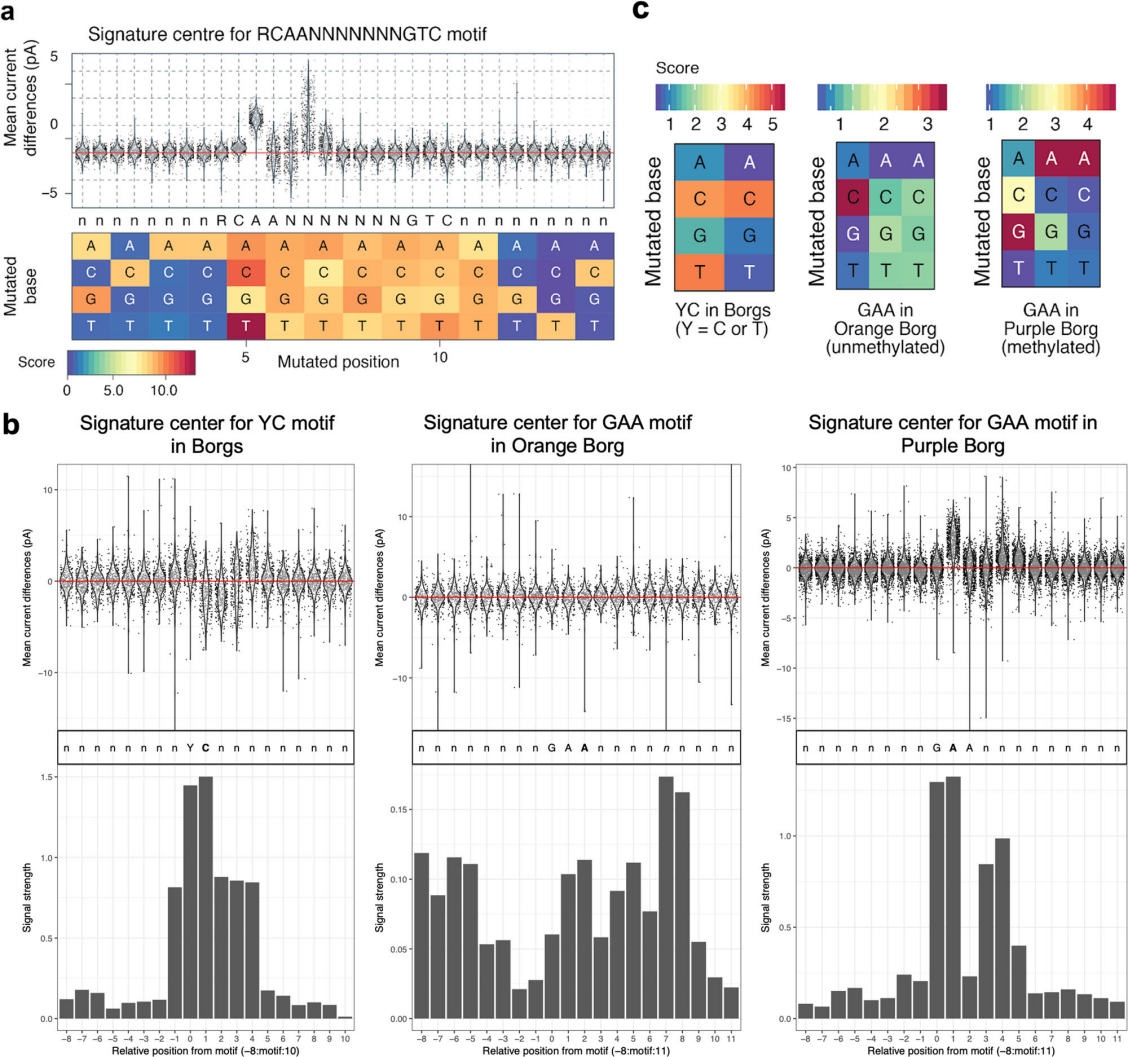
*Methanoperedens* and Borgs have distinct methylation patterns. **a** Current differences between native and amplified DNA at RCAANNNNNNNGTC motif sites in cMp. **b** Current differences between native and amplified DNA at YC motif sites in Borgs, GAA motifs in Orange Borg, and GAA in Purple Borg. **c** Motif refinement plot for related motifs with one substitution. The score represents a current difference score where blue represents no current difference (nonmodified base) and red a large current difference (modified base).

Given abundance ratio variation and some very large predicted Borg copies per most abundant potential host genome, as well as tentative indications of viral proteins and genes involved in encapsulation, we hypothesized that Borgs and *Methanoperedens* have distinct methylation patterns to distinguish between self and non-self DNA. Thus, we searched for DNA methylation motifs in the nanopore datasets by comparing the mean current of native DNA vs. MDA-amplified DNA using Nanodisco. This brought to light nine methylation motifs in the *Methanoperedens* genome, six of which were substantiated by read-level methylation calling with Megalodon using its models for N^6^ adenine-specific and 5-methylcytosine-specific DNA methylations (6mA and 5mC) detection (Fig. 6a, Supplementary Data 12). Performing the same analysis on the genomes of Orange, Ochre, Black, and Purple Borg revealed that they have a modification motif composed of YC, in which the cytosine is methylated and the Y stands for either pyrimidine T or C. Surprisingly, these motifs are found 184-192 times per 1 kbp of Borg genome, and they are particularly dominant (71%) on the non-coding strand of each replichore. Modification of cytosine residues was previously detected in the 72 kbp linear dsDNA genome of the STSV1 virus infecting *Sulfolobus tengchongensis* where it is proposed to protect the viral DNA from degradation through host machinery, and ensure selective regulation of viral replication and transcription^29^. Since it is a dinucleotide, the YC modification motif is reminiscent of CpG sites that are found in eukaryotes, including DNA viruses infecting eukaryotes. In eukaryotic viruses, the CpG sites are important in regulating gene expression, but also serve an intricate regulatory role during the viral life cycle^30^. Purple Borg has an additional motif composed of GAA, which happens to be the complement of the TC motif (Fig. 6b, c, Supplementary Data 12).

Based on models for methylation detection, we infer that the GAA motif has a 6 mA modification in the first A, and we speculate that the YC motifs contain a 4mC modification. This 4mC modification may be introduced by the DNA methylase that is found in all Borgs (Supplementary Fig. 5). This is supported by the finding that a homolog is also encoded in Sulfolobus virus STSV1 (YP_077254.1) and STSV2 (YP_007348303.1), and interestingly also in the Haloarcula virus HJTV-2 (YP_010357641.1). The Borg host genomes bMp and cMp encode several restriction-modification systems (types I, II, and III), which could also target Borg DNA at some point in their existence. We conclude that *Methanoperedens* and Borg genomes have clearly distinct methylation patterns, and these could be important in regulating Borg replication and gene expression.

### Widespread Borg OmcZ nanowire genes that enhance metabolism

Recently, methane-consuming archaea were proposed to use nanowires to transfer metabolic electrons to extracellular electron acceptors, such as minerals or syntrophic partners (extracellular electron transfer, EET)^31–34^. However the Borg proteins identified are not known to transfer electrons or play a role in metabolism^34^. Subsequently, it was noted that *Methanoperedens* genomes encode nanowire assembly genes homologous to cytochrome OmcZ, which is processed by OzpA (OmcZ protease), and prolyl isomerase, and EET has been confirmed experimentally^31,35^. Of 111 cytochromes, OmcZ is the only nanowire-forming outer-surface cytochrome essential for long-range (>10 µm) EET by microbial communities^31,32^.

**Fig. 7:**
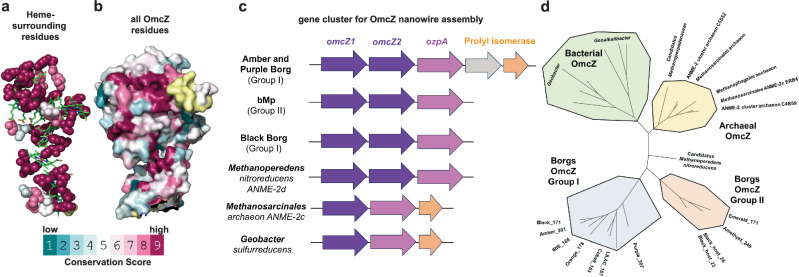
OmcZ nanowire assembly genes that enhance metabolic capacity are widespread in Borgs. Evolutionary conservation of **a**. residues within 3 Å from the heme chain and **b**. surface residues of OmcZ (PDB 7lq5). Yellow regions represent residues lacking enough sequences for comparison. **c**. The nanowire assembly gene cluster is conserved across diverse Borgs and their hosts. **d** Phylogenetic tree based on OmcZ nanowire assembly proteins.

Importantly, we identified potential OmcZ genes in Borgs and evaluated the likelihood that they form nanowires by analyzing conserved structural and assembly features. There is high conservation of the electron-transferring heme region (Fig. 7a), including the histidine pair that brings hemes closer to confer the 1000-fold higher conductivity of OmcZ compared to other nanowires^31,32^ (Supplementary Fig. 7a).

Both the predicted OmcZ of Borgs and co-occurring *Methanoperedens* show high conservation of key surface residues that confer high protein stability in denaturing and acidic (pH <1.6) conditions^31,32^ (Fig. 7b). A key feature of OmcZ proteins is the heme-binding motifs. Non-canonical heme-binding motifs contain more than two residues between the cysteine residues (CXnCH, *n* > 2), whereas the canonical heme-binding motif contains exactly two residues (CXXCH). The structure of OmcZ nanowires from *Geobacter sulfurreducens* showed a non-canonical heme-binding motif (CX_14_CH) with additional residues forming a solvent-exposed loop^31^. *G. sulfurreducens* OmcZ can reduce diverse soluble electron acceptors, and removing this loop suppressed the ability of OmcZ to reduce them, suggesting this loop could be trapping soluble electron acceptors^31^. In contrast to *G. sulfurreducens* OmcZ, a subset of Borg OmcZ proteins did not show this non-canonical heme-binding motif. Thus, the host *Methanoperedens* could use their OmcZ-like nanowires to pass electrons to insoluble, rather than soluble, electron acceptors. The OmcZ-precursor in Black Borg has two additional β-strand enriched domains compared to bacterial OmcZs^31,32^ (Supplementary Fig. 7b–d), but the significance of these remains uncertain.

Notably, the homologs of OmcZ form two clades, the first composed only of Borgs (Group I) and the second composed of the *Methanoperedens* hosts and other Borgs (Group II). Both clades are distinct from all other OmcZ homologs (Fig. 7d). The entire gene cluster required for OmcZ nanowire assembly, comprising OmcZ and the expected protease and isomerase, is conserved in several Borgs and in their host’s genomes (Fig. 7c, d, Supplementary Fig. 8). As reconstitution of this cluster in a heterologous host is sufficient to produce OmcZ that self-assembles into nanowires^31^, our findings suggest that *Methanoperedens* could use OmcZ nanowires encoded by Borg genomes to enhance their metabolic capacity to couple methane oxidation to reduction of soil-associated terminal electron acceptors, such as ferric iron oxides or oxyhydroxides.

### Metabolic genes of *Methanoperedens* and Borgs are expressed in situ

**Fig. 8:**
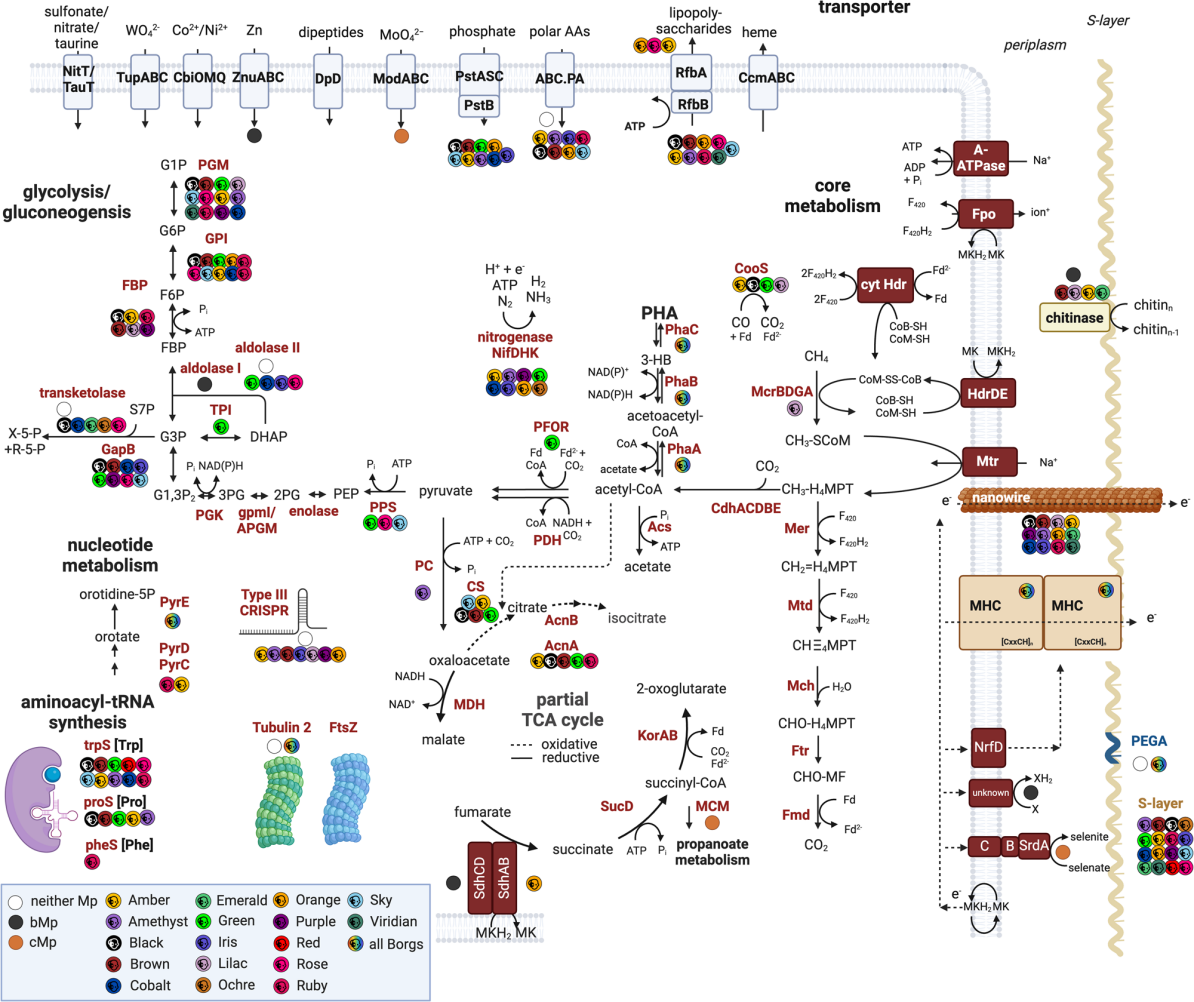
Metabolic reconstruction of two *Methanoperedens* that host Borgs and metabolic potential of Borgs. Most proteins depicted are present in both *Methanoperedens* genomes. Proteins absent in one of the two or both *Methanoperedens* genomes are accompanied by a black, orange, or white circle. Proteins present in Borg genomes are accompanied by colored circles with a Borg cartoon symbol. For a more detailed explanation, refer to the circle and color legend at the bottom of the figure and for a detailed list of abbreviations and locus tags, please refer to Supplementary Data 14. Created with BioRender.com.

Reconstructing the metabolism of the genomes of *Methanoperedens* that are predicted Borg hosts confirmed that their main metabolism is likely anaerobic methane oxidation (Fig. 8, Supplementary Data 1^1^). Other than terminal electron acceptor reactions mediated by nanowires, electrons from methane oxidation may be donated to selenate reductase in cMp. Borgs encode a plethora of protein homologs that facilitate an expansion of the host-encoded metabolic genes, including CO dehydrogenase, glycolysis, and carbohydrate-related genes, transporters involved in the export of lipopolysaccharides, or the import of phosphate and polar amino acids (Fig. 8, Supplementary Data 14). Both *Methanoperedens* and Borgs also encode numerous S-layer proteins that likely build the outermost layer of the cell. PEGA-domain proteins are additional surface proteins that are exclusively found in Borgs, with four to 21 genes largely co-localized in an individual Borg genome (Supplementary Data 2, Supplementary Data 14).

We then performed full-length cDNA and gDNA nanopore sequencing on newly collected wetland soil samples (two samples from 50 cm, one sample from 90 cm, 100 cm, 115 cm) in search of transcripts indicating that Borgs and *Methanoperedens* were active at the time of sample collection. Analysis of the metagenomic dataset revealed that these samples consistently contained *Methanoperedens* bMp and Black Borg but no other Borgs. Mapping the cDNA reads revealed that only 8–16% of bMp genes and ≤1% of cMp genes had transcripts detected (Supplementary Data 15a–c). The Mcr genes of *Methanoperedens* were most highly expressed (≤225×), followed by genes (≤17×) encoding cell architecture and electron transport-mediating proteins, including the S-layer protein, tubulin/FtsZ, adhesion proteins, a phasin, MHCs and nanowire proteins (Supplementary Data 16).

Surprisingly, we detected transcripts for 45–57% of the genes encoded in Black Borg genome (Supplementary Data 16). The nanowire-forming OmcZ genes were among the highest expressed genes of Black Borg. Assuming the inferred Black Borg - bMp linkage is correct, the genomic ratio of Black Borg and its host bMp ranged from 0.4:1 to 0.9:1 in the three samples (Supplementary Data 17). Notably, the Borg transcript abundance for the nanowire protein ranged from 3:1 to 6:1 compared to bMp transcripts, indicating that the Borg-encoded nanowire protein is more highly expressed than the host-encoded protein. Other genes in Black Borg with well-detected transcripts were other MHCs, a membrane-embedded glycosyltransferase, and core proteins of unknown function (subfam1116 and subfam2007). Overall, this metatranscriptomic analysis illuminated that *Methanoperedens* and Borg genes are expressed simultaneously and that membrane-anchored and extracellular Borg proteins contribute to cell architecture and terminal redox transfer.

### Conserved Borg proteins enable the discovery of new Borgs

The existence of marker proteins distributed over most of the Borg genomes opened the way for improved identification of new Borgs. Previously, we used ribosomal protein L11A (rpL11), a near-marker Borg protein, to locate and distinguish Borgs. A SingleM search identified one publicly available dataset from 1.5 m depth in a peatland ecosystem warming experiment in Northern Minnesota, USA^36^, with reads encoding parts of rpL11 from three novel Borgs. Thus, we searched for a new assembly of this metagenome with the 40 marker protein sequences and identified 37 to 40 homologs for three Borgs named Maroon (~65× coverage), Mauve (~65× coverage), and Liserian (~17× coverage). The Maroon contigs could be distinguished from those of Mauve as the marker proteins are similar to those of Sky and Rose Borgs, whereas Mauve and Liserian proteins are related to those of other Borgs, including Amethyst and Ruby Borgs. Maroon Borg marker protein-bearing contigs, along with candidate contigs with the expected GC content, phylogenetic profile, and coverage, were manually curated into a 634 kbp near-complete genome with two gaps (originally 11 contigs), ordered based on synteny with the Sky Borg genome. The genome shares all features with previously defined Borgs but displays much fewer TR. Intriguingly, there is no clear indication of *Methanoperedens* in this sample, raising the possibility that Borgs replicate in the methanogenic archaea that are present. Alternatively, *Methanoperedens* may be at very low abundance or the Borgs may exist as environmental DNA, protected from degradation by encapsulation or another mechanism.

### Borg marker gene phylogeny and the “tree of Borgs”

**Fig. 9:**
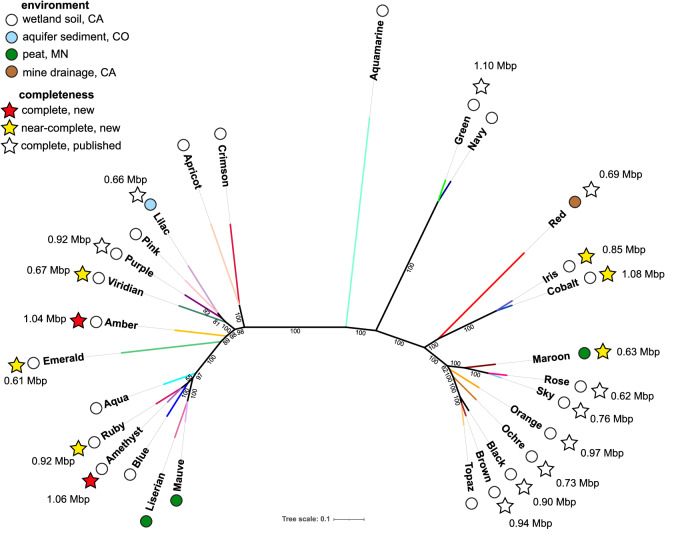
A phylogenetic tree showing the relatedness of 28 Borgs, their genome sizes, completeness, and environment of origin. 40 Borg marker proteins were aligned, trimmed, and concatenated into a supermatrix containing 9185 amino acid positions of marker proteins originating from 28 Borgs. Bootstraps above 55 are displayed.

We constructed phylogenetic trees for each of the 40 marker proteins, including homologs from bins for uncurated (or partially curated) Borgs and the 17 complete/near-complete Borg genomes. The trees all indicate similar evolutionary relationships amongst the Borgs, including the subdivision of most Borgs into two distinct clades. Given this, we concatenated the 40 sequences of proteins from all Borgs for which we could identify at least 60% of homologs of the marker proteins with reasonably high confidence (in the case of draft genomes). In total, the concatenated marker protein sequences represent 28 different Borgs. The phylogenetic tree generated from the alignment of these sequences confirms the existence of two major clades, one of which was previously represented only by fragments in bins and two complete genomes (Fig. 9).

This research substantially expands what we know about huge Borg extrachromosomal elements of anaerobic methane-oxidizing *Methanoperedens* archaea. Relative to other ECEs, including plasmids and huge phages, Borgs carry a remarkable inventory of genes for metabolism and functions related to genome stability and replication. Conserved and generally syntenous Borg single-copy marker genes define a shared genomic backbone. Phylogenetic analyses indicate vertical inheritance from a common ancestral type, followed by diversification into two clades. One conserved marker gene encodes an origin of replication binding protein, and other members of the core proteome are predicted to respond to DNA damage and maintain chromosome integrity. We demonstrate that multiheme cytochromes involved in the terminal electron transfer from methane metabolism are ubiquitous and always present in multiple copies, strengthening the inference that Borgs have evolved mechanisms to augment their host’s ability to oxidize methane. In fact, metatranscriptomic data reveal that Black Borg putative nanowires are expressed more highly than those of their putative host *Methanoperedens*.

Although Borgs share many genes, few are ubiquitous, consistent with a propensity for gene acquisition via lateral transfer, enabling huge flexibility in gene content. While at first glance resembling plasmids, Borg genomes may encode capsid proteins and seem to have features shared with viruses, including double-stranded linear DNA viruses of eukaryotes. The 17 well-defined Borg genomes display a consistent genome architecture, further supporting the classification of Borgs as a specific and seemingly novel type of genetic element. Finally, we present the first evidence that distinct methylation patterns may enable *Methanoperedens* to distinguish self vs. Borg genomes. Thus, drawing solely on curated metagenomic and metatranscriptomic sequence information, this study provides a glimpse into the nature and existence of enigmatic genetic elements of some of the most interesting and arguably biogeochemically important archaea yet known.

## Methods

### Sampling and nucleic acid extraction

We recovered deep soil samples from 75 to 120 cm below the soil surface from a wetland in Lake County, California, USA, in September 2021 to construct new metagenomic datasets. DNA was extracted from 5-10 g of soil per sample with the DNeasy PowerMax Soil Kit (Qiagen) and used for short-read and long-read sequencing. The same site was sampled again at four different depths (twice at 50 cm, once at 90 cm, 100 cm, and 115 cm) in November 2022 to construct metatranscriptomic and matching metagenomic datasets. RNA and DNA was co-extracted using a combination of the RNeasy PowerSoil Total RNA Kit and the RNeasy PowerSoil DNA Elution Kit (Qiagen), and then further processed for long-read nanopore sequencing (see below).

### Whole-cell extraction DNA crosslinking, and viral concentration

Whole cells were extracted from soil samples by separating particles through low-speed centrifugation and subsequent density centrifugation with Optiprep (Sigma). Half of the cell pellet suspension was subjected to formaldehyde treatment (1% v/v) to crosslink protein and nucleic acid within whole cells, and the other half served as a control cell suspension. DNA was extracted and used for long-read sequencing. Borg DNA from Black Borg was detected in control cells, but the crosslinked samples did not provide sufficient recovery for sequencing and further analysis. Another attempt to enrich Borgs was undertaken by supersaturating wetland soil with potassium citrate buffer, sequential particle separation through low-speed centrifugation three to five times, and passing the supernatant through a 0.22 µm membrane and Amicon filter. DNA extracted from the filtrate was sequenced and used in the correlation analysis described in this study.

### Short-read and long-read sequencing for metagenomics

Paired-end 2 × 250 bp reads were generated from Illumina sequencing on a NovaSeq SP 250PE at the QB3 facility, University of California, Berkeley, USA. Sequencing adapters, PhiX, and other Illumina trace contaminants were removed with BBTools^37^ (v38.79), and sequence trimming was performed with Sickle^38^ (v1.33). Filtered reads were assembled with IDBA-UD^39^(v1.1.3) or SPAdes^40^(v3.15.4), ORFs were predicted with Prodigal^41^(v2.6.3) and functionally annotated by comparison to KEGG, UniRef100 and UniProt using USEARCH^42^(v10.0.240).

Long-read sequencing was performed on three samples using GridION and PromethION P24 sequencing devices at Oxford Nanopore Technologies Inc., New York, USA. Native DNA libraries were prepared with the Ligation Sequencing Kit (LSK114). MDA-amplified DNA libraries were prepared with the Repli-G mini kit (Qiagen) and LSK114. Libraries were loaded onto either Oxford Nanopore Technologies’ FLO-MIN106D (R9.4.1), FLO-PRO002 (R9.4.1), FLO-MIN114 (R10.4.1), or FLO-PRO114M (R10.4.1) flowcells, and sequenced for 72 h with super accuracy basecalling. Shorter reads (<1 kb) or low-quality reads (mean *q* score ≤ 10) were filtered out with fastp^43^(v0.23.4). Adapter sequences were removed using porechop (v0.2.4)^44^. Branching artifacts caused by multiple displacement amplification were removed by aligning reads to themselves with mappy^45^(v.2.24), followed by the removal of reads with non-diagonal self-alignment. Native and MDA-amplified reads from R10.4.1 flowcells were jointly assembled with metaFlye^46^(v2.9). Contigs were polished with medaka consensus (v1.7.1). Short-read polishing was done with Hapo-G^47^(v1.3.1).

### Long-read sequencing and data processing for metatranscriptomics

Total RNA samples were polyadenylated using *Escherichia coli* poly(A) polymerase (NEB, M0276L) for 5 min and purified by GeneJET RNA Cleanup and Concentration Micro Kit (ThermoFisher, K0841). The poly(A)-tailed total RNA samples were reverse transcribed and PCR-amplified into full-length cDNA following the Oxford Nanopore Technologies PCR cDNA Synthesis (PCS109) protocol. cDNA amplicons were barcoded (EXP-NBD114), pooled and sequenced with FLO-PRO114M flowcells on PromethION, and basecalled using Guppy (v6.3.9). Adapter sequences were removed using porechop, and reads were trimmed with bbduk (minavgquality=20 qtrim=rl trimq=20). The cDNA reads were then mapped onto the 17 curated Borg genomes and two *Methanoperedens* genomes using minimap2 (-ax map-ont --secondary=no)^45^(v.2.24-r1122). Alignments were filtered using seqkit bam (--field ReadCov –range-min 70, –field Acc –range-min 95). The alignments per gene were calculated with featureCounts (--fracOverlapFeature 0.1)^48^ (v2.0.3).

### Manual genome curation

Illumina reads were mapped to nanopore-based assembled sequences classified to be of Borg origin to assess the overall sequence accuracy. Manual curation was undertaken with the goal of generating complete genomes. Obviously, chimeric regions, often evident by a large change in GC content and by lack of Illumina read support, as well as unsupported sequence blocks that consisted of mirror images, were removed. Regions lacking perfect read support (0 SNPs allowed) throughout the sequence were flagged and, where possible, errors corrected by mapping reads with lower stringency (e.g., 3% SNPs) to the flagged regions. Individual base pairs in the consensus sequence were then inserted/deleted/replaced manually. Internal segments without read support were removed and replaced by gap filling informed by Illumina reads and using Illumina contigs that were identified by blastn. As Illumina contigs can be chimeric and contain local assembly errors, curation benefited from the availability of Illumina-assembled sequences from multiple samples. The majority of Illumina sequences were favored where there was uncertainty, and the newly incorporated sequence was verified by stringent Illumina read mapping. Contig ends were extended using unplaced Illumina reads until newly incorporated sequences were sufficient to recruit additional Illumina or nanopore contigs. Assembled sequence ends were extended into terminal ITRs by read mapping until no further extension was possible. Given the generally high read coverage throughout, this was taken to signify the ends of the genome. The genome was then considered complete, so long as there was perfect Illumina read support throughout. In two cases where it was not possible to generate a single Borg sequence extended into ITRs, contigs were ordered and oriented based on alignment with complete Borg genomes. The completeness of genomes not fully extended was assessed based on replichore structure (two replichores of unequal length anticipated), cumulative GC skew, and alignment with complete genomes.

### Tandem repeat identification, replication prediction, and genome topology verification

Tandem repeats were identified using a custom Python script (https://github.com/rohansachdeva/tandem_repeats)^6^. Nucleotide tandem repeats were searched using a stringent threshold of ≥50 nt regions and ≥3 TR units and allowing no mismatch (—min_region_len 50—min_repeat_count 3). Amino acid tandem repeats were identified in the concatenated proteins file using a threshold of ≥16 amino acids and ≥3 TRs (-l 3—min_repeat_- count 3—min_uniq_nt 1—min_region_len 16). GC skew (G - C/G + C) and cumulative GC skew were calculated to identify the replication origin and termini, and verify genome topology using the iRep package (gc_skew.py)^49^.

### Structural modeling and structure-based homology search

Structural modeling of all Orange Borg proteins was performed using AlphaFold2^8^ via a LocalColabFold (—use_ptm—use_turbo—num_- relax Top5—max_recycle 3)^50^. The AF2 rank1 models were profiled against the Protein Data Bank (http://www.rcsb.org/pdb/) (v5e639, date: 2023-04-20) using foldseek^51^ (v53465) and the easy-search module, or manually queried in PDBeFold^52^. Protein structures were visualized and superimposed in PyMOL^53^ (v2.3.4).

### Protein family clustering and functional annotation of protein subfamilies

A dataset of 20,280 Borg proteins was constructed using all 8322 protein-encoding ORFs of the 7 new Borg genomes, and the 11,958 Borg proteins from the previously published 10 complete Borg genomes^6^. All proteins were clustered into protein subfamilies using MMseqs2^54^ (v7e284) in an all-vs.-all search (e-value: 0.001, sensitivity: 7.5, and cover: 0.5). A sequence similarity network was built based on pairwise similarities, and protein subfamilies were defined using the greedy set-cover algorithm. HHblits^55^(v3.0.3) was used to construct Hidden-Markov models (HMMs) for these protein subfamilies based on the results2msa of MMseqs2. These were then profiled against the PFAM database using HHsearch^56^ (v3.0.3) and an HMM-HMM comparison.

### Functional annotation of individual proteins

Proteins were profiled using InterProScan^57^ (v5.51-85.0) and HMMER (hmmer.org) (v3.3, hmmsearch) against the PFAM (--cut_nc) and KOFAM (--cut_nc) HMM databases^58,59^. TMHs were predicted with TMHMM^60^ (v2.0) and cellular localization using PSORT^61^ (v2.0, archaeal mode). tRNAs were searched with tRNAscan^62^ (v.2.0.9) and rRNAs with SSU-ALIGN^63^ (v0.1.1). Protein subfamilies were functionally annotated using HMMER (hmmer.org) (v3.3, hmmsearch) and the PFAM (--cut_nc) HMM database^62^. Homology search was performed with blastp^57^ against the NCBI database and our own database in ggkbase (https://ggkbase.berkeley.edu/). Metabolic reconstruction was based on the Distilled and Refined Annotation of Metabolism (DRAM) framework^58^ (v1.2.2) and on custom annotations.

### Correlation analysis

To measure correlations between abundance patterns of Borgs and potential *Methanoperedens* hosts, a dataset was compiled consisting of genomic sequences from both Borg and *Methanoperedens* genomes in addition to phylogenetically distinct unbinned contigs containing rpL11 genes profiled as *Methanoperedens* as per Al-Shayeb et al. 2022. These sequences were aligned to 83 metagenomic samples obtained from the wetland soil site. Alignments were performed using bbmap.sh^37^ using the following parameters: ambiguous = random minid = 0.96 idfilter = 0.97. For samples sequenced with 150 bp reads, the editfilter = 5 parameter was used; for those samples sequenced with 250 bp reads, editfilter=8 was used. Reads aligning to each genome or rpL11-bearing contig were counted and used to calculate average coverage for each. Coverage values were normalized by sequencing depth by calculating the total number of base pairs sequenced per sample and dividing coverage by this value, then multiplying by a 1e11 scaling factor. Pearson correlations between sequencing depth-normalized coverage patterns for each genome or rpL11 contig across all 83 samples were then calculated using pandas and scipy in python (v3.8.2). Clustering and visualization were performed using seaborn, matplotlib, and scipy; code for visualization was generated with the assistance of GPT-4^59^.

### Detection of Borg and *Methanoperedens* DNA modification patterns

Methylation motifs and type predictions were found using Nanodisco^60^, which identifies loci with high contrast between the ionic current levels of the native and multiple displacement amplification datasets. The R9.4.1 reads were used for methylation analysis since Nanodisco does not support the newer R10 data. Although the Borg motifs were too short to be identified directly, Nanodisco identified several that contained CC, TC, or GAA. The true motifs were narrowed down to YC and GAA by manual inspection of Nanodisco refinement plots, which indicated that additional bases were extraneous.

Independently, methylation was called on individual reads with Megalodon, using Rerio models for all-context 6mA and 5mC modifications. However, the YC motif was not found with this method, supporting Nanodisco’s predicted modification type of 4mC. An all-context 4mC Rerio model does not currently exist.

### Genome visualization and alignments

Genomes were visualized in Geneious Prime 2021.2.2 and aligned with the MCM algorithm, or progressiveMauve for aligning multiple contigs. Gene neighborhood analysis was performed with clinker^61^(v0.0.21).

### Taxonomic classification

16 S rRNA gene sequences were used for taxonomic classification of archaeal genomes (Methanoperedens, Methanomicrobiales). The 16 S rRNA genes were identified using a custom HMM^63^ (16SfromHMM.py, available at GitHub https://github.com/christophertbrown/bioscripts) and taxonomically classified using the SILVA database^64^. Taxonomic classification was also routinely performed for all genes as part of an integrated pipeline in ggkbase. The gene annotations are compared to the uniprot and uniref databases and taxonomically classified by the best match in these databases.

### Marker-based identification of a Borg-containing peat metagenome

SingleM^65^ (v0.13.2) was used to identify rpL11 sequences from an in-house database of public metagenomes (published June 2019 or earlier) that was scanned using the SingleM “query” subcommand using --max-divergence 3, using rpL11 OTU sequences derived from Borg reference genomes. This identified the run SRR7028199 as potentially containing Borg genomes. Draft Borg genome bins were identified based on shared low GC, coverage, and dominance by novel proteins without phylogenetic affiliations. The bin for the Borg genome that was curated to completion was refined by alignment to the Sky Borg genome and for contigs carrying the Borg marker genes.

### Phylogenetic tree construction

Individual phylogenetic trees were constructed using the protein sequences for the 40 marker proteins to confirm concordant topologies, consistent with vertical inheritance. Blastp searches used the 40 marker proteins to identify homologs in additional Borgs (for which complete and near-complete genomes were not available) in the wetland soil and SPRUCE peat data. The single-marker datasets were aligned with MAFFT^66^ (v7.481) using the option L-INS-I, and alignments were trimmed using BMGE^67^ (v1.12) with the BLOSUM30 substitution matrix. Trimmed alignments were then concatenated into a character supermatrix totaling 9,185 amino acid positions and 28 Borgs. A maximum likelihood tree was then built from this supermatrix with IQ-TREE^68^ (v1.6.12) using the mixture model LG + C60 + F + R4 with ultrafast bootstrap support^69^ calculated from 1000 replicates and visualized iTOL^70^.

### Bioinformatic analysis of Borgs OmcZ homologs

Protein phylogeny tree of nanowire-forming OmcZ was constructed by maximum likelihood method with MEGA X^71^ and presented by iTOL^70^. Nanowire-forming OmcZ sequences were extracted from full-length OmcZ homologs by removing signal peptide and self-inhibitory part after the subtilisin cleavage site. Signal peptide cleavage sites were predicted by SignalP^72^ (5.0). The *ozpA* cleavage sites were chosen at the corresponding positions to the OzpA cleavage site in *G. sulfurreducens* OmcZ (after FGNSS) in the multisequence alignment. OmcZ homologs without signal peptides were used for AlphaFold2^8^ modeling. The conservation of OmcZ homologs, which were aligned by Clustal Omega^73^, was mapped to the structure of nanowire-forming OmcZ (PDB: 7lq5) by Consurf^74^ and presented by PyMOL^53^ (v2.3.4).

### Reporting summary

Further information on research design is available in the Nature Portfolio Reporting Summary linked to this article.

### Supplementary information

Supplementary Information

Peer Review File

Description of Additional Supplementary Files

Supplementary Data 1–17

Reporting Summary

## Data Availability

Newly released Borg and *Methanoperedens* genomes used in this manuscript are available via: https://ggkbase.berkeley.edu/borgs_mp_nanopore/organisms and have been deposited in the NCBI database under accession code PRJNA1119519. The publicly available datasets used in this study are available on NCBI under PRJNA866293 and PRJNA914281. The SPRUCE dataset is accessible under GOLD Project ID# Gp0213362 (https://gold.jgi.doe.gov/search). Protein sequences, structural models, and the phylogenetic tree of the 28 Borgs from this study are available through Zenodo (10.5281/zenodo.8162866). Supplementary Information including Supplementary Figs. and detailed annotations and larger datasets are available in Supplementary Figures 1–8 and Supplementary Data 1–17.
