# Supplementary material for: Borg extrachromosomal elements of methane-oxidizing archaea have conserved and expressed genetic repertoires
Source: Nat Commun. 2024 Jun 26;15:5414. doi: 10.1038/s41467-024-49548-8 (PMC11208441; doi:10.1038/s41467-024-49548-8)
Supplement: Supplementary file 1 — Supplementary Information [file 41467_2024_49548_MOESM1_ESM.pdf]

Supplementary Figures for:

Borg extrachromosomal elements of methane-oxidizing archaea have conserved and expressed genetic repertoires

Marie C. Schoelmerich[1*], Lynn Ly[2], Jacob West-Roberts[3], Ling-Dong Shi[1], Cong Shen[4,5], Nikhil S. Malvankar[4,5], Najwa Taib[6], Simonetta Gribaldo[6], Ben J. Woodcroft[7], Christopher W. Schadt[8,9], Basem Al-Shayeb[1], Xiaoguang Dai[2], Christopher Mozsary[2], Scott Hickey[2], Christine He[2], John Beaulaurier[2], Sissel Juul[2], Rohan Sachdeva[1], and Jillian F. Banfield[1,3,10,11#]

[1]Innovative Genomics Institute, University of California, Berkeley, CA, USA
[2]Oxford Nanopore Technologies Inc, New York, NY, USA
[3]Department of Environmental Science, Policy and Management, University of California, Berkeley, CA, USA
[4]Microbial Sciences Institute, Yale University, New Haven, CT, USA
[5]Deptartment of Molecular Biophysics and Biochemistry, Yale University, New Haven, CT, USA
[6]Institut Pasteur, Université de Paris cité, Unit Evolutionary Biology of the Microbial Cell, Paris, France
[7]Centre for Microbiome Research, School of Biomedical Sciences, Queensland University of Technology (QUT), Translational Research Institute, Woolloongabba, Australia
[8]Biosciences Division, Oak Ridge National Laboratory, Oak Ridge, TN, USA
[9]Department of Microbiology, University of Tennessee, Knoxville, TN, USA
[10]Biomedicine Discovery Institute, Monash University, Vic, Australia
[11]Department of Earth and Planetary Science, University of California, Berkeley, CA, USA
*current affiliation: Department of Environmental Systems Sciences, ETH Zurich, 8092 Zurich, Switzerland

[#] Corresponding author: jbanfield@berkeley.edu

This PDF file includes:

Supplementary Figures 1 – 8 and legends

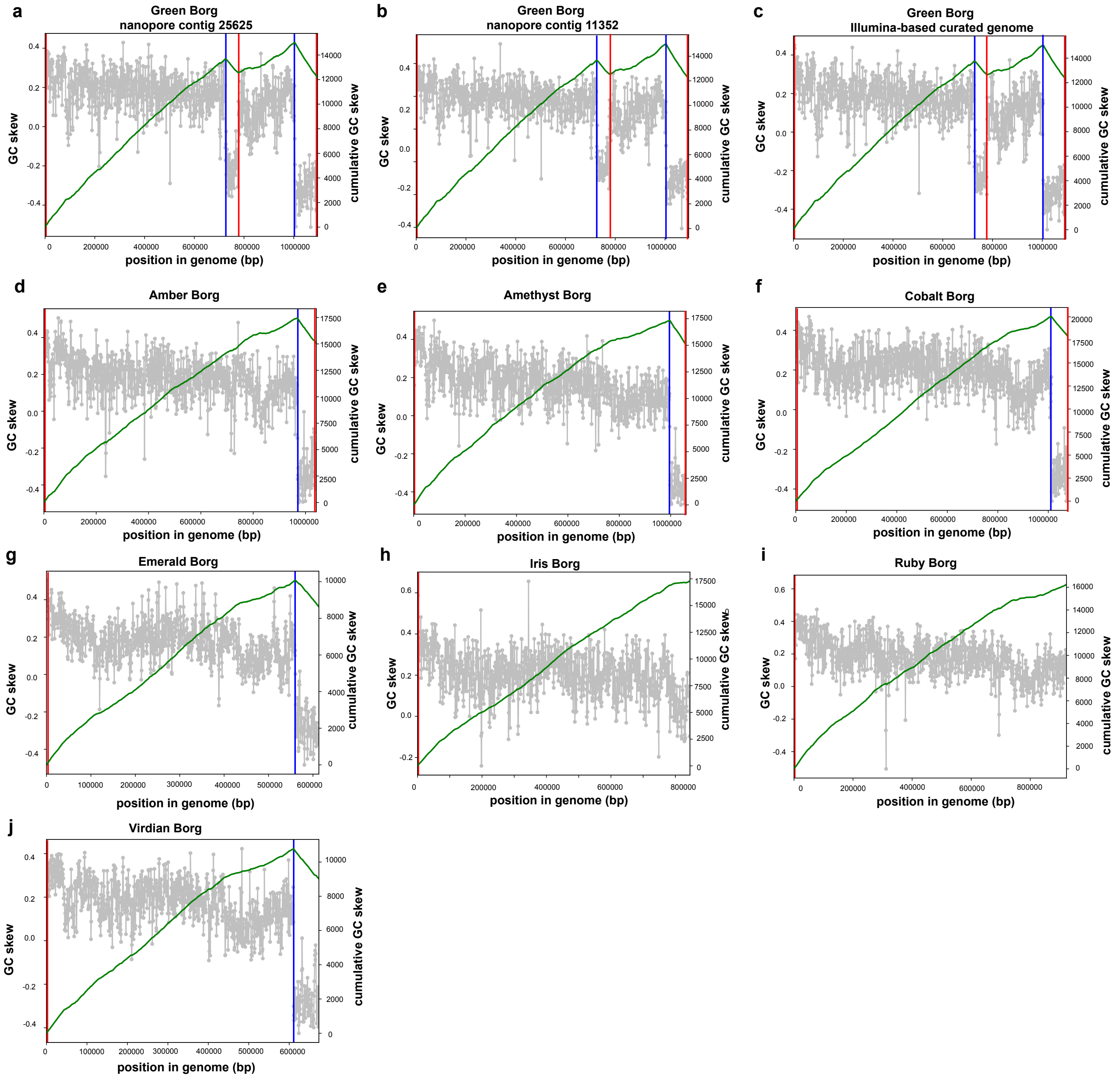

Supplementary Figure 1. GC skew analysis of nanopore-derived Green Borg contigs and Illumina-based curated genome, and GC skew of new Borg genomes. Green Borg contigs derived from nanopore assemblies were recovered in two different soil samples and the GC skew plots confirm the overall consistent topology (**a**, **b**) that was established based on manually curated Illumina assemblies only (**c**, already published in [1]). Recovery of 7 new Borg genomes from the nanopore assemblies shows a consistent (cumulative) GC skew for these genomes (**d-j**), suggesting replication from the termini. Iris, Ruby, and Viridian Borg are not complete, as reflected by the missing drop in the GC skew peak in the cumulative GC skew, which is absent due to the missing small replichore.

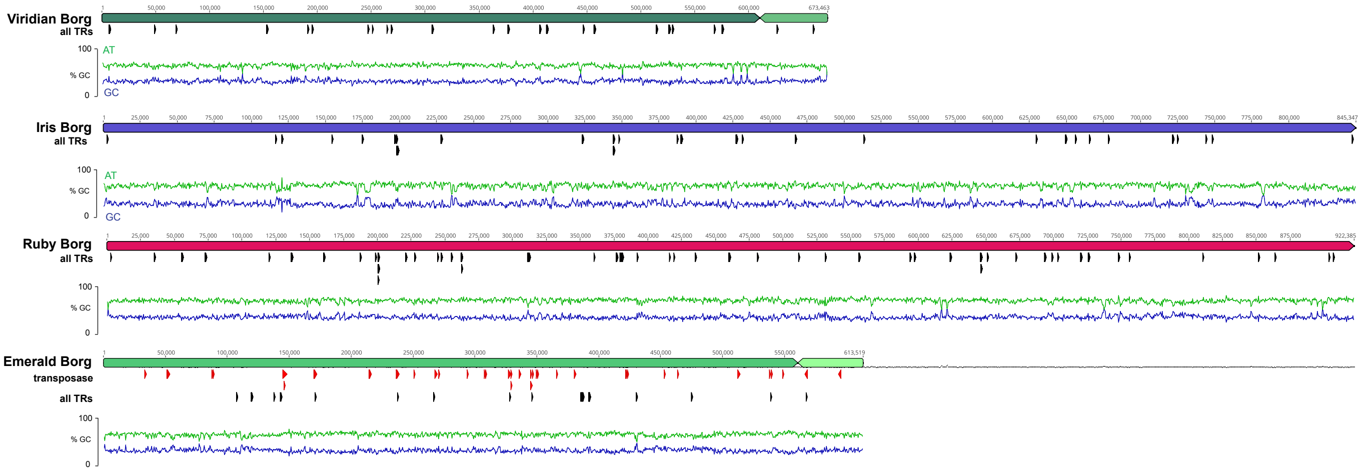

Supplementary Figure 2. Overviews of the non-complete but manually curated Borg genomes. Red markings indicate the locations of numerous transposase genes in the Emerald genome.

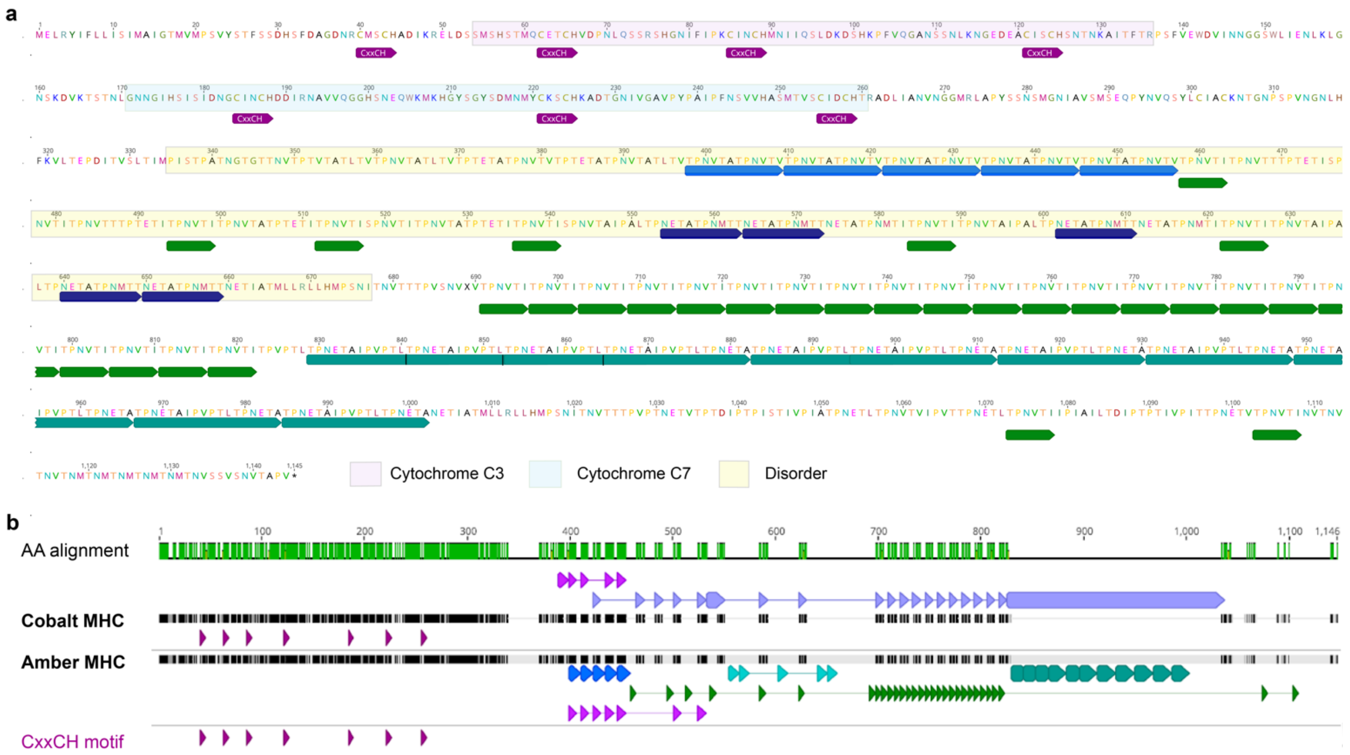

Supplementary Figure 3. Repetitive regions in Borg DNA. a. Amber Borg genome encodes a multi-heme cytochrome (7 heme-binding motifs: CxxCH) that is a complex repeat-bearing protein. Colored arrows with the same color indicate the same repeat sequence. **b**. Alignment of the Amber MHC with TR with that of the Amethyst MHC with TR. Note that a portion of some unit repeats in the two proteins are shared.

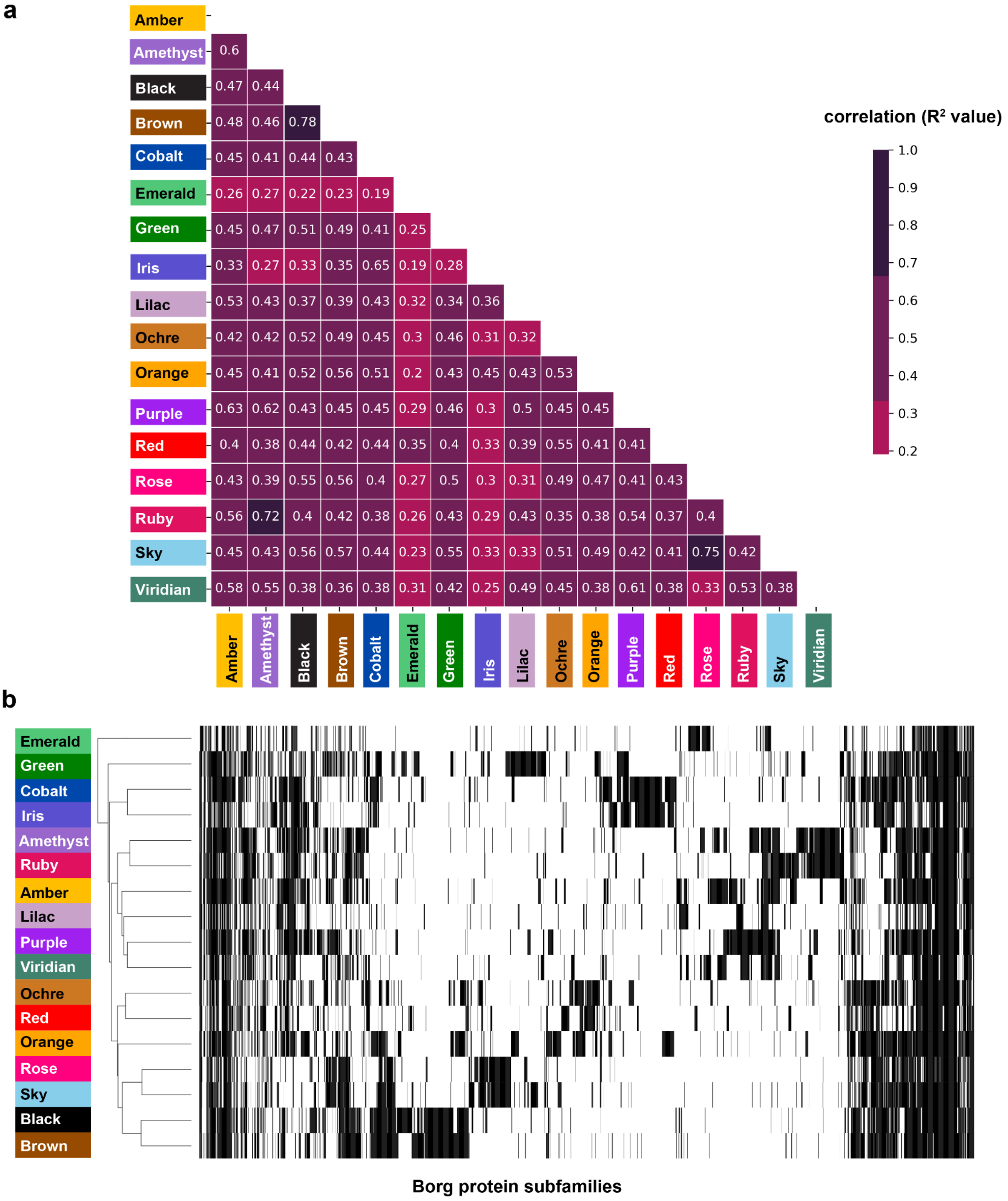

Supplementary Figure 4. Borg proteome content. **a**. Shared protein subfamily correlation analysis reveals proteome-based relatedness of the 17 curated complete and near-complete Borgs. **b**. Heatmap showing presence (black) or absence (white) of protein subfamily members in the 17 Borgs. Borgs sharing more protein subfamilies cluster together (i.e., Black and Brown Borg).

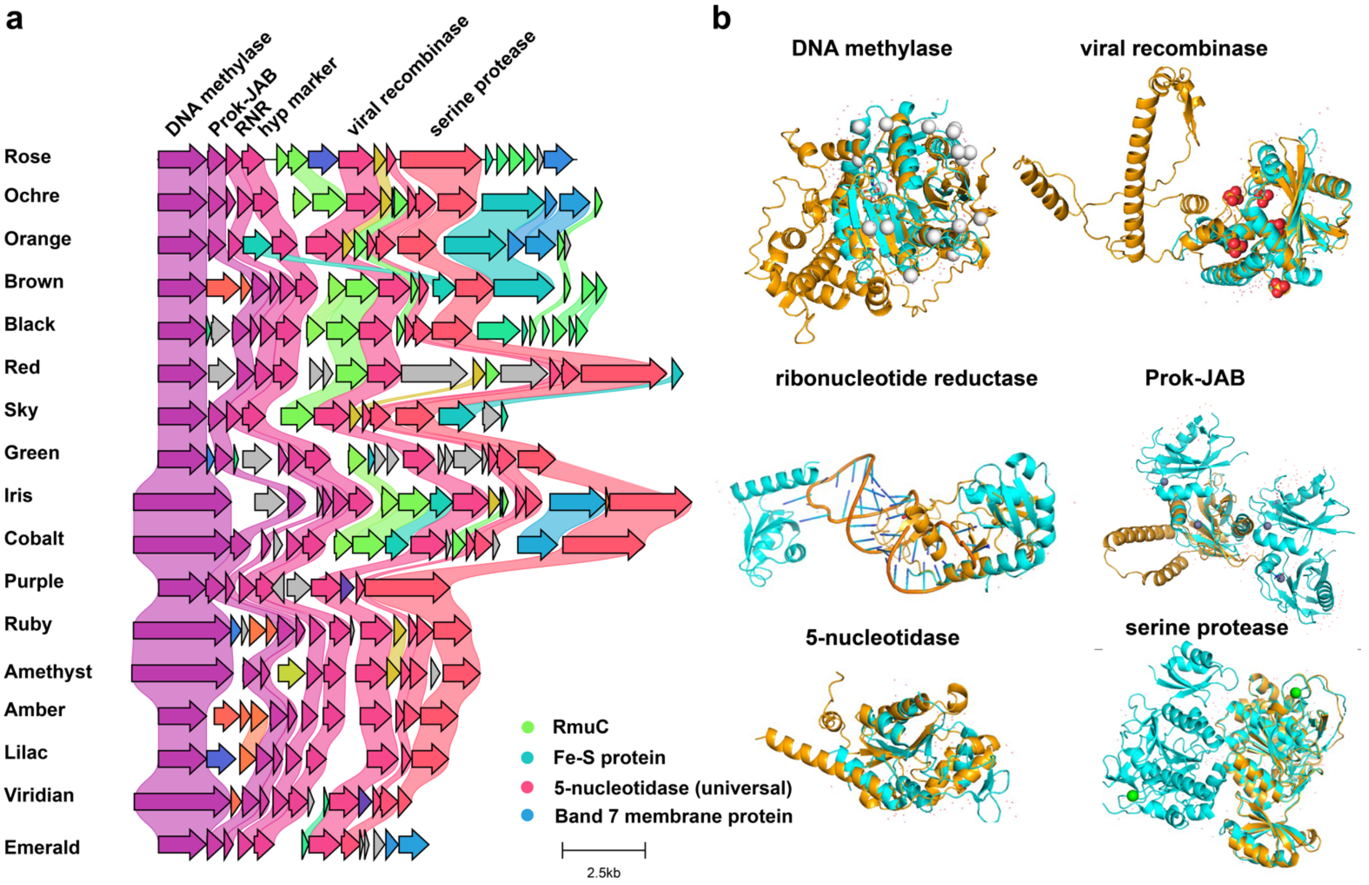

Supplementary Figure 5. **Conserved genomic region in all 17 Borgs comprises nucleotide processing machinery. a**. Colored genes highlight homologs based on amino acid identity. The alignment was generated with clinker using the default settings. **b**. Protein structures from Orange Borg were predicted with AF2 and are shown in Orange. Best structural hits were obtained through PDBefold search [2]. The best structural hit for DNA methylase is human NMRT-1 (PDB match 5e1b, RMSD = 2.81), for the viral recombinase is lambda exonuclease (PDB match 6m9k, RMSD = 1.84), for the ribonucleotide reductase (RNR) it is the RNA-binding XRRM domain of human LARP7 (PDB match 6d12, RMSD = 2.06), for the Prok-JAB it is AF2198, a JAB1/MPN domain protein from *Archaeoglobus fulgidus* (PDB match 1oi0, RMSD = 3.32), for the 5-nucleotidase it is a magnesium dependent phosphatase 1 (MDP-1; PDB match 1u7o, RMSD = 2.42), for the serine protease it is IS1-inserted Pro-subtilisin E (PDB match 3whi, RMSD = 1.03).

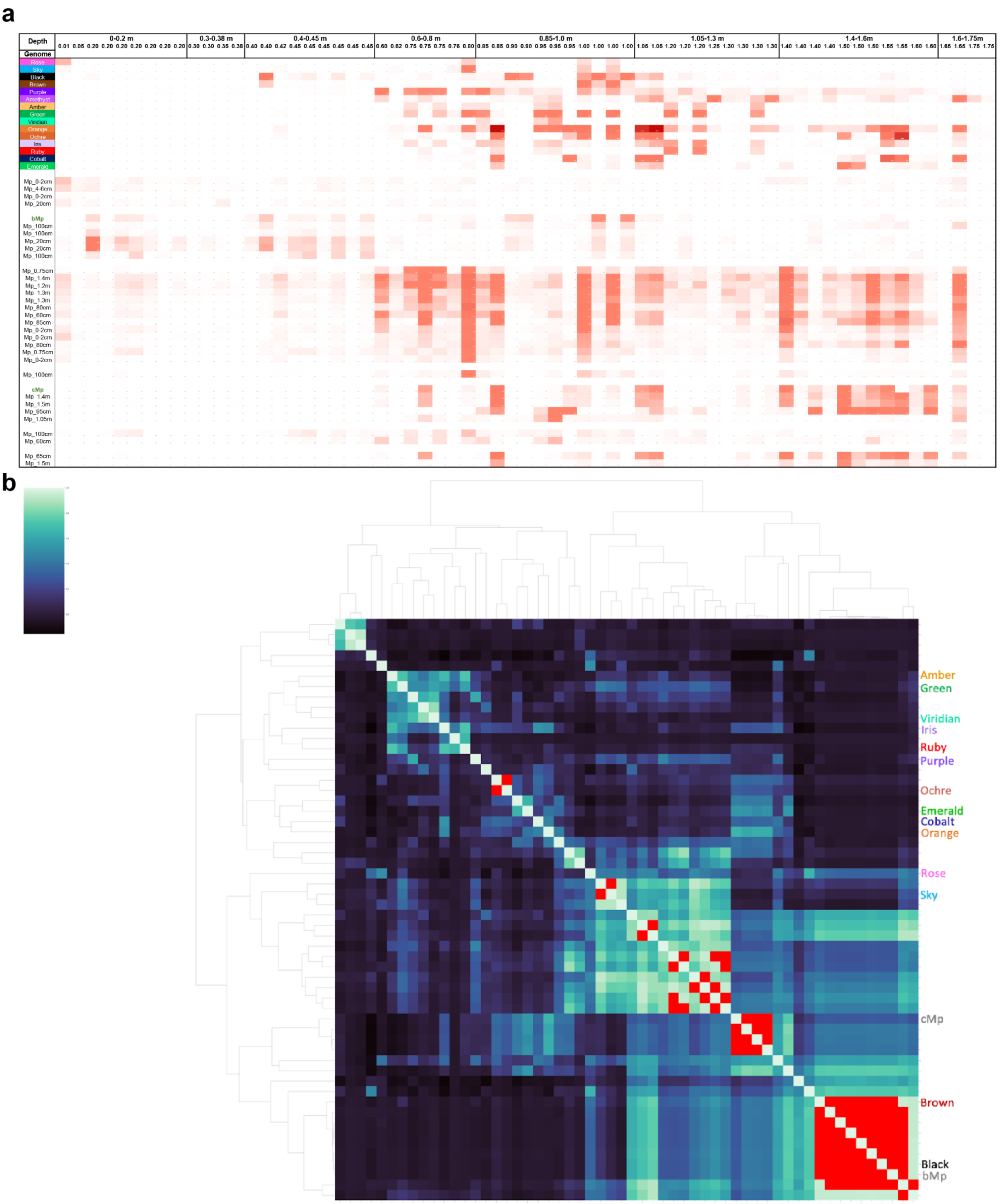

Supplementary Figure 6. Borg and *Methanoperedens* species abundances over samples, normalized for sequencing depth. a. Normalized abundance over samples and depth. The full table can be found in **Supplementary Table 7. b.** Correlation analysis between coverage patterns of *Methanoperedens* genomes, population representative *Methanoperedens* rpL11 contigs, and Borg genomes across 83 samples. Red cells indicate correlation scores above 0.95.

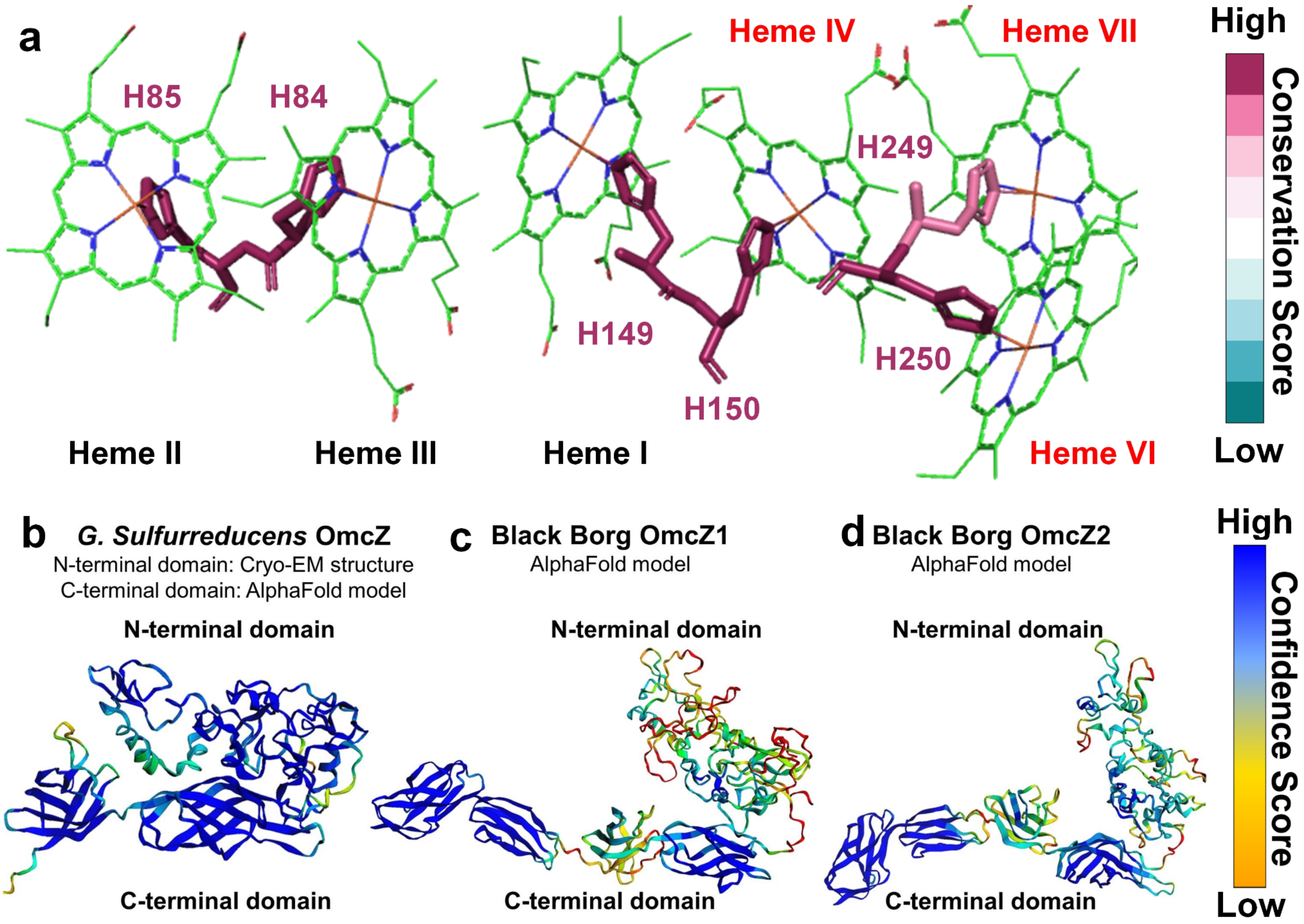

Supplementary Figure 7. Key structural features important for electron transfer in OmcZ nanowires show high evolutionary conservation in Borgs and hosts. a. Histidine pairs that lock hemes more tightly to confer high nanowire conductivity are highly conserved in Borgs and hosts. The residue numbering follows the amino acid sequence of Black Borg OmcZ1 and includes the signal peptide. **b.** AlphaFold model predicts with high confidence that bacterial OmcZ-precursor shows only two β-strand enriched domains in the C-part whereas **c.** OmcZ1- and **d.** OmcZ2- precursors from Black Borgs show four β-strand-enriched domains in the C-part.

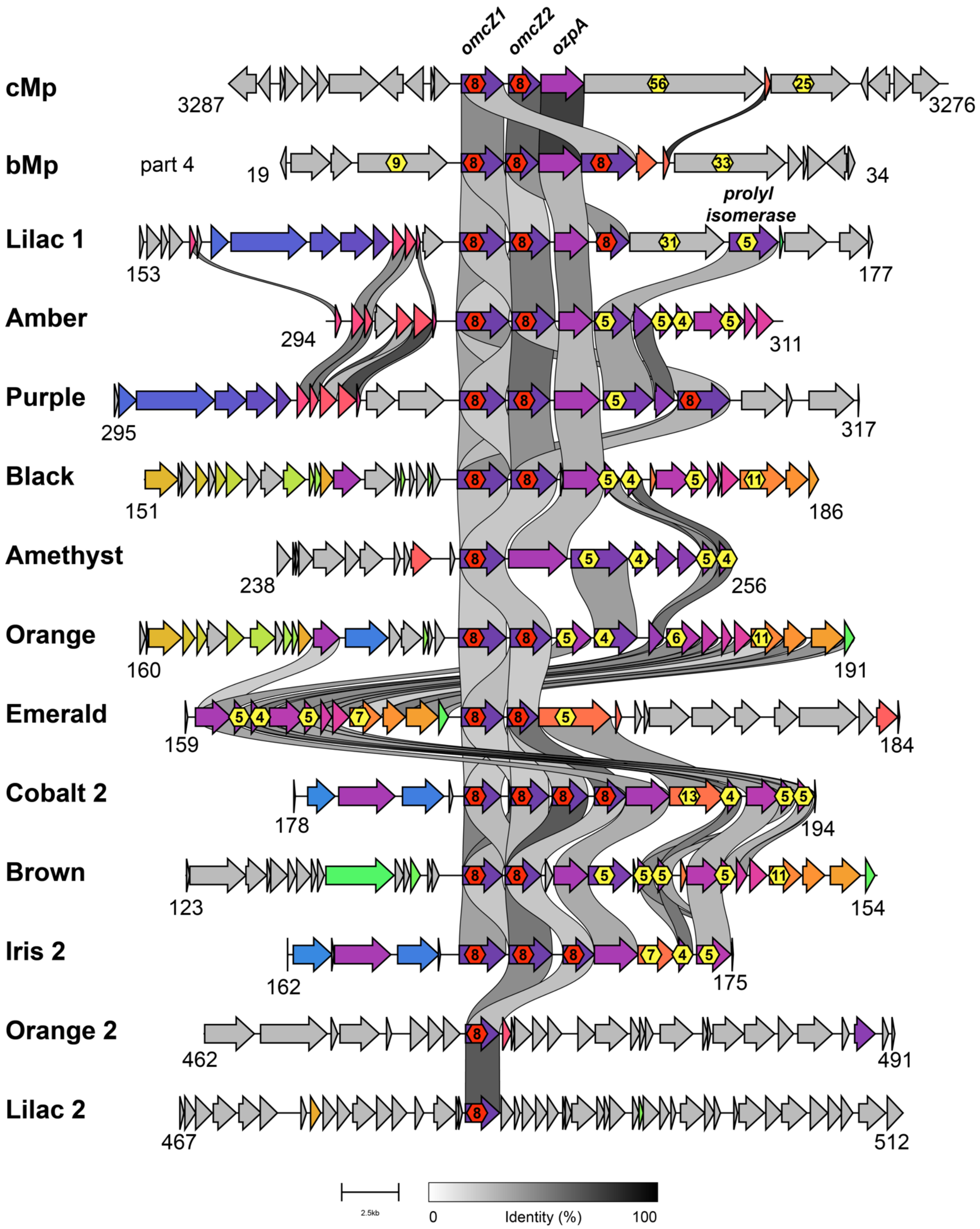

Supplementary Figure 8. **Genomic neighborhood alignments of potential nanowire-forming OmcZ clusters in Borgs and *Methanoperedens***. Alignments were performed with clinker and gene coordinates are shown. Number of heme-binding motifs are depicted in yellow or red hexagons.

Supplementary References

1. Schoelmerich, M. C., Sachdeva, R., West-Roberts, J., Waldburger, L. & Banfield, J. F. Tandem repeats in giant archaeal Borg elements undergo rapid evolution and create new intrinsically disordered regions in proteins. *PLoS Biol.* **21**, e3001980 (2023).
2. Krissinel, E. & Henrick, K. Secondary-structure matching (SSM), a new tool for fast protein structure alignment in three dimensions. *Acta Crystallogr. D Biol. Crystallogr.* **60**, 2256–2268 (2004).
