# Supplementary material for: Borg extrachromosomal elements of methane-oxidizing archaea have conserved and expressed genetic repertoires
Source: Nat Commun. 2024 Jun 26;15:5414. doi: 10.1038/s41467-024-49548-8 (PMC11208441; doi:10.1038/s41467-024-49548-8)
Supplement: Supplementary file 3 — Description of Additional Supplementary Files [file 41467_2024_49548_MOESM3_ESM.docx]

**Description of Additional Supplementary Files for:**

**Borg extrachromosomal elements of methane-oxidizing archaea have conserved and expressed genetic repertoires**

**Supplementary Data Legends**

**File Name: Supplementary Data 1.**

**Description: Taxonomic classification of all Borg proteins based on uniprot and uniref comparisons.**

**File Name: Supplementary Data 2.**

**Description: Annotations and features of all proteins encoded in the 17 Borgs.**

**File Name: Supplementary Data 3.**

**Description: Protein subfamilies consistently encoded in Borgs.** Marker and near-marker proteins: exactly one example occurs in all 17 or 16/17 Borgs, respectively, listed in order of their location in the Black Borg genome. Multicopy marker proteins occur in all genomes but occur in >1 copy in at least one Borg genome. Protein subfamilies were numbered (1-40 marker proteins; 1*-22* near marker proteins; M1-M24 multicopy proteins). The colors indicate colocalization of the proteins in several Borg genomes.

**File Name: Supplementary Data 4.**

**Description: MHC proteins in Borgs, bMp and cMp.** All proteins with >= 3 CxxCH motifs are listed here.

**File Name: Supplementary Data 5.**

**Description: Protein subfamilies established from the 17 Borgs, cMp and bMp.** This protein clustering was performed to establish if Borgs share proteins with cMp or bMp.

**File Name: Supplementary Data 6.**

**Description: Statistically significant correlations in the pattern of abundance over samples for Borgs.** Coverage values for Borg genomes, *Methanoperedens* scaffolds carrying rpL11, the cMp and bMp genomes and draft *Methanoperedens* genomes across 65 soil samples were used to detect possible Borg-host linkages. The data support the prior inference that Black Borg replicates in the bMp genome and suggest that Brown Borg may also replicate in this or a related species.

**File Name: Supplementary Data 7.**

**Description: Normalized abundance of Borgs and *Methanoperedens* species across 70 samples.** All samples originate from the wetland soil in Lake County, CA, USA. Fifteen of the 17 Borgs central to this study, as well as cMp and bMp were recovered from these samples.

**File Name: Supplementary Data 8.**

**Description: Capsid-related structural matches of Orange Borg proteins from AF2-modeled protein structures.**

**File Name: Supplementary Data 9.**

**Description: CheckV output of 17 Borg genomes.** End_to_end pipeline was run using v1.0.1 (Nayfach *et al.,* 2021).

**File Name: Supplementary Data 10.**

**Description: GeNomad output of 17 Borg genomes.** End_to_end pipeline was run using v1.5.1 (Camargo *et al.*, 2023) and *contigs_virus_genes* table is shown.

**File Name: Supplementary Data 11.**

**Description: DNA replication machinery of Herpesvirus and putative functional homologs in Borgs.** The table is based on Weller & Coen, 2012, and was populated with Borg proteins from this study.

**File Name: Supplementary Data 12.**

**Description: Methylation motif and frequency in the cMp genome and Borgs.**

**File Name: Supplementary Data 13.**

**Description: Annotations and features of all proteins encoded in cMp and bMp.**

**File Name: Supplementary Data 14.**

**Description: List of elements shown in the metabolic reconstruction of *Methanoperedens* and Borgs depicted in Figure 8.** Annotations are based on custom annotations for Borg and *Methanoperedens* proteins (Supplementary Data 2 & 13), and output of DRAM. For a list of MHCs see Supplementary Data 4, and nanowire genes, see Supplementary Data 16.

**File Name: Supplementary Data 15a.**

**Description: Summary of metatranscriptomic analysis.** All cDNA reads were mapped on 17 Borg genomes, and the two *Methanoperedens* genomes bMp and cMp.

**File Name: Supplementary Data 15b.**

**Description: Number of features per genome.**

**File Name: Supplementary Data 15c.**

**Description: Percentage of genes with transcript.**

**File Name: Supplementary Data 16.**

**Description: Metatranscriptomic data of Borgs and *Methanoperedens* from four soil samples recovered from a wetland site in Lake County, CA.** Columns H-L show the number of reads that mapped to the respective ORF.

**File Name: Supplementary Data 17.**

**Description: Coverage of Black Borg and bMp in nanopore metagenomic dataset originating from the same samples as the metatranscriptomic dataset.** The DNA samples from 100cm and 115cm depth did not yield sufficient yields for reliable sequencing.
