# Supplementary material for: Borg extrachromosomal elements of methane-oxidizing archaea have conserved and expressed genetic repertoires
Source: Nat Commun. 2024 Jun 26;15:5414. doi: 10.1038/s41467-024-49548-8 (PMC11208441; doi:10.1038/s41467-024-49548-8)
Supplement: Supplementary file 5 — Reporting Summary [file 41467_2024_49548_MOESM5_ESM.pdf]

nature portfolio

Corresponding author(s): Jill Banfield

Last updated by author(s): June 4, 2024

Reporting Summary

Nature Portfolio wishes to improve the reproducibility of the work that we publish. This form provides structure for consistency and transparency in reporting. For further information on Nature Portfolio policies, see our Editorial Policies and the Editorial Policy Checklist.

Statistics

For all statistical analyses, confirm that the following items are present in the figure legend, table legend, main text, or Methods section.

n/a	Confirmed	
☐	☒	The exact sample size (*n*) for each experimental group/condition, given as a discrete number and unit of measurement
☐	☒	A statement on whether measurements were taken from distinct samples or whether the same sample was measured repeatedly
☐	☒	The statistical test(s) used AND whether they are one- or two-sided<br>*Only common tests should be described solely by name; describe more complex techniques in the Methods section.*
☐	☒	A description of all covariates tested
☐	☒	A description of any assumptions or corrections, such as tests of normality and adjustment for multiple comparisons
☐	☒	A full description of the statistical parameters including central tendency (e.g. means) or other basic estimates (e.g. regression coefficient) AND variation (e.g. standard deviation) or associated estimates of uncertainty (e.g. confidence intervals)
☒	☐	For null hypothesis testing, the test statistic (e.g. *F*, *t*, *r*) with confidence intervals, effect sizes, degrees of freedom and *P* value noted<br>*Give P values as exact values whenever suitable.*
☒	☐	For Bayesian analysis, information on the choice of priors and Markov chain Monte Carlo settings
☐	☒	For hierarchical and complex designs, identification of the appropriate level for tests and full reporting of outcomes
☐	☒	Estimates of effect sizes (e.g. Cohen's *d*, Pearson's *r*), indicating how they were calculated

Our web collection on statistics for biologists contains articles on many of the points above.

Software and code

Policy information about availability of computer code

Data collection

Samples were collected from a wetland in California, USA.
DNA was extracted with the DNeasy PowerMax Soil Kit (Qiagen).
DNA and RNA were co-extracted with the RNeasy PowerSoil Total RNA Kit and the RNeasy PowerSoil DNA Elution Kit (Qiagen).
DNA libraries were generated by the QB3 facility, Berkeley, USA.
Short reads (paired end 2x250 bp) were generated from Illumina sequencing on a NovaSq SP 250PE.
Native DNA libraries were prepared with the Ligation Sequencing Kit (LSK114). MDA-amplified DNA libraries were prepared with the Repli-G mini kit (Qiagen) and LSK114.
Long reads were generated from GridION and PromethION P24 sequencing devices at Oxford Nanopore Technologies.
Data was prepared and analyzed using the following tools:
Geneious Prime (v2021.2.2; licensed, paid version used in this study, free versions available)
BBTools (v38.79)
Sickle (v1.33)
IDBA-UD (v1.1.3)
SPAdes (3.15.4)
FastP (v0.23.4)
Porechop (v0.2.4)
Mappy (v.2.24)
metaFlye (v2.9)
medaka (v1.7.1)

Hapo-G (v1.3.1)
Guppy (v6.3.9)
minimap2 (v.2.24-r1122)

Data analysis

Python (v.3.8.3)
MCM algorithm in Geneious Prime (v2021.2.2)
progressiveMauve in Geneious Prime (v2021.2.2)
Prodigal (v2.6.3)
USEARCH (v10.0.240)
FeatureCounts (v2.0.3)
InterProScan (v5.51-85.0)
hmmsearch (v3.3)
tRNAscan (v.2.0.9)
SSU-ALIGN (v0.1.1)
AlphaFold2
LocalColabFold (v1.2.0)
PyMOL (v2.3.4)
clinker (v0.0.21)
MMseqs2 (v7e284)
HHblits (v3.2.0)
HHsearch (v3.2.0)
foldseek (v53465)
GC skew analysis with code on github (https://github.com/christophertbrown/iRep/tree/master/iRep)
DRAM (v1.2.2)
Nanodisco for methylation motif and type prediction
Megalodon using Rerio models for methylation calling
SingleM (v0.13.2)
MEGA X
MAFFT (v7.453)
IQ-TREE (v1.6.12)
BMGE (v1.12)
iTOL
SignalP (v.5.0)
Clustal Omega (https://www.ebi.ac.uk/jdispatcher/msa/clustalo)
Consurf (https://consurf.tau.ac.il/consurf_index.php)

For manuscripts utilizing custom algorithms or software that are central to the research but not yet described in published literature, software must be made available to editors and reviewers. We strongly encourage code deposition in a community repository (e.g. GitHub). See the Nature Portfolio guidelines for submitting code & software for further information.

Data

Policy information about availability of data

All manuscripts must include a data availability statement. This statement should provide the following information, where applicable:

- Accession codes, unique identifiers, or web links for publicly available datasets
- A description of any restrictions on data availability
- For clinical datasets or third party data, please ensure that the statement adheres to our policy

Newly released Borg and Methanoperedens genomes used in this manuscript are available via: https://ggkbase.berkeley.edu/borgs_mp_nanopore/organisms and have been deposited in the NCBI database under accession code PRJNA1119519. The publicly available datasets used in this study are available on NCBI under PRJNA866293 and PRJNA914281. The SPRUCE dataset is accessible under GOLD Project ID# Gp0213362 (https://gold.jgi.doe.gov/search). Protein sequences, structural models, and the phylogenetic tree of the 28 Borgs from this study are available through Zenodo (10.5281/zenodo.8162866). Supplementary Information including Supplementary Figures and detailed annotations and larger datasets are available in Supplementary Figures 1-8 and Supplementary Tables 1-17.

Research involving human participants, their data, or biological material

Policy information about studies with human participants or human data. See also policy information about sex, gender (identity/presentation), and sexual orientation and race, ethnicity and racism.

Reporting on sex and gender: not applicable

Reporting on race, ethnicity, or other socially relevant groupings: not applicable

Population characteristics: not applicable

Recruitment	not applicable
Ethics oversight	not applicable

Note that full information on the approval of the study protocol must also be provided in the manuscript.

Field-specific reporting

Please select the one below that is the best fit for your research. If you are not sure, read the appropriate sections before making your selection.

☒ Life sciences ☐ Behavioural & social sciences ☐ Ecological, evolutionary & environmental sciences

For a reference copy of the document with all sections, see nature.com/documents/nr-reporting-summary-flat.pdf

Life sciences study design

All studies must disclose on these points even when the disclosure is negative.

Sample size

14 environmental samples were collected from a wetland site and are listed in NCBI under SUB14504770.
The samples were chosen to provide a high breadth of ecosystem coverage for the recovery of Borg genomes. The following 9/14 samples were used for shotgun metagenomics using Illumina sequencing:
SR-VP_9_9_2021_87_5B_1_2m_2
SR-VP_9_9_2021_49_3B_1_65m
SR-VP_9_9_2021_65_4B_1_25m
SR-VP_26_10_2020_2_100CM
SR-VP_9_9_2021_59_4A_0.85m
SR-VP_9_9_2021_72_4B_1.05m
SR-VP_9_9_2021_75_5A_0.95m
SR-VP_9_9_2021_81_5A_0.75m
SR-VP_9_9_2021_87_5B_1.2m
The following 9/14 samples were used for Nanopore sequencing :
SR-VP_9_9_2021_72_4B_1.05m for whole genomes sequencing (WGS) r10, MDA r10 and WGS r9 and MDA r9 for methylation calling with Nanodisco
SR-VP_9_9_2021_75_5A_0.95m for WGS r9
SR-VP_9_9_2021_81_5A_0.75m for WGS r10, MDA r10
SR-VP_9_9_2021_87_5B_1.2m for WGS r10, MDA r10
SR-VP_2022_11_27_#0-1_50cm WGS r10 for matching metatranscriptomic data
SR-VP_2022_11_27_0-2_50cm WGS r10 for matching metatranscriptomic data
SR-VP_2022_11_27_2_90cm WGS r10 for matching metatranscriptomic data
SR-VP_2022_11_27_3_115cm WGS r10 for matching metatranscriptomic data
SR-VP_2022_11_27_P_100cm WGS r10 for matching metatranscriptomic data
The following samples were used for DNA/RNA coextractions and subsequent cDNA analyses:
SR-VP_2022_11_27_#0-1_50cm
SR-VP_2022_11_27_0-2_50cm
SR-VP_2022_11_27_2_90cm
SR-VP_2022_11_27_3_115cm
SR-VP_2022_11_27_P_100cm

Data exclusions

DNA samples from 100cm and 115cm depth did not yield sufficient yields for reliable nanopore sequencing and were thus excluded from coverage calculations shown in Supplementary Table 15.

Replication

Borg genomes were recovered from multiple independent samples collected in 2021 and 2022. The samples were all collected at the same location, yet soil heterogeneity prevents taking biological replicates. Nevertheless, the same Borgs and Methanoperedens genomes were recovered in multiple samples from this same site, allowing for a verification of the genome sequences.

Randomization

Randomization is not applicable for the environmental samples.
Protein family clustering was performed to evaluate which proteins are single copy marker proteins of Borgs.

Blinding

Investigators were not blinded to group allocation during data analysis in this study. Initial investigatory analysis of the data required the investigators to know the true groupings of the data to understand the results of data clustering and dimension reduction performed at the onset of the analysis.

Reporting for specific materials, systems and methods

We require information from authors about some types of materials, experimental systems and methods used in many studies. Here, indicate whether each material, system or method listed is relevant to your study. If you are not sure if a list item applies to your research, read the appropriate section before selecting a response.

Materials & experimental systems

n/a	Involved in the study
☒	☐ Antibodies
☒	☐ Eukaryotic cell lines
☒	☐ Palaeontology and archaeology
☒	☐ Animals and other organisms
☒	☐ Clinical data
☒	☐ Dual use research of concern
☒	☐ Plants

Methods

n/a	Involved in the study
☒	☐ ChIP-seq
☒	☐ Flow cytometry
☒	☐ MRI-based neuroimaging

Plants

Seed stocks	not applicable
Novel plant genotypes	not applicable
Authentication	not applicable
